# PI4P-mediated solid-like Merlin condensates orchestrate Hippo pathway regulation

**DOI:** 10.1126/science.adf4478

**Published:** 2024-08-09

**Authors:** Pengfei Guo, Bing Li, Wei Dong, Huabin Zhou, Li Wang, Ting Su, Christopher Carl, Yonggang Zheng, Yang Hong, Hua Deng, Duojia Pan

**Affiliations:** 1 Department of Physiology, Howard Hughes Medical Institute, University of Texas Southwestern Medical Center; Dallas, TX 75390, USA.; 2 Department of Cell Biology, University of Pittsburgh; Pittsburgh, PA 15261, USA.; 3 Department of Biophysics, Howard Hughes Medical Institute, University of Texas Southwestern Medical Center; Dallas, TX 75390, USA.

## Abstract

Despite recent studies implicating liquid-like biomolecular condensates in diverse cellular processes, many biomolecular condensates exist in a solid-like state, and their function and regulation are less understood. We show that the tumor suppressor Merlin, an upstream regulator of the Hippo pathway, localizes to both cell junctions and medial apical cortex in *Drosophila* epithelia, with the latter forming solid-like condensates that activate Hippo signaling. Merlin condensation required phosphatidylinositol-4-phosphate (PI4P)-mediated plasma membrane targeting, and was antagonistically controlled by Pez and cytoskeletal tension through plasma membrane PI4P regulation. The solid-like material properties of Merlin condensates are essential for physiological function and protect the condensates against external perturbations. Collectively, these findings uncover an essential role for solid-like condensates in normal physiology and reveal regulatory mechanisms for their formation and disassembly.

## One-Sentence Summary

A tumor suppressor forms solid-like condensates to regulate Hippo signaling

Liquid-liquid phase separation (LLPS) drives the formation of biomolecular condensates that regulate diverse cellular processes (1–5). Despite the widespread occurrence of dynamic liquid-like condensates, many biomolecular condensates exist in a non-dynamic, solid-like state (3, 5–7). These include protein deposits in pathological conditions such as FUS and other prion-like RNA-binding proteins (RBPs) in amyotrophic lateral sclerosis (ALS) and Frontotemporal dementia (FTD) (reviewed in 5), as well as condensates that perform their normal cellular function in a solid-like state, such as centrosomes (8), nuclear pores (9) and *oskar* ribonucleoprotein granules (10). In contrast to liquid-like condensates, the function and regulation of solid-like condensates in normal cell physiology are poorly understood.

The Hippo signaling pathway controls tissue growth and its malfunction contributes to human diseases including cancer (11–15). This pathway was first characterized in *Drosophila* and involves a core kinase cascade comprising the Hippo (Hpo)-Salvador (Sav) kinase complex, the Warts (Wts)-mob as tumor suppressor (Mats) kinase complex, and the Scalloped (Sd)-Yorkie (Yki) transcription complex. The Hippo core kinase cascade is in turn regulated by several upstream regulators that relay diverse inputs into the pathway, such as mechanical tension, cell polarities, osmotic stress and cell-cell contact (11–15). The versatility of the Hippo pathway to respond to diverse inputs distinguishes it from other developmental pathways such as Wnt, Hedgehog, Transforming growth factor beta (TGFβ) and Notch pathways, which are all activated by pathway-specific ligands. Although such versatility is thought to underlie an organism’s exquisite ability to fine tune tissue growth with tissue architecture, the mechanisms by which these upstream regulators integrate diverse inputs remain to be fully elucidated.

The Hippo pathway upstream inhibitor Sarcolemmal membrane-associated protein (SLMAP) and the upstream activators angiomotin (AMOT) and kidney and brain expressed protein (KIBRA) form functionally antagonizing biomolecular condensates via LLPS, and the coalescence of these functionally antagonizing, liquid-like condensates into a common condensate drives Hippo pathway activation by osmotic stress and cell-cell contact (16). In the current study, we focused on Merlin, another upstream regulator of Hippo signaling (17–20) and a classic tumor suppressor whose mutations underlie the human disease Neurofibromatosis Type II (NF2) (21). We found that, in contrast to the other upstream regulators of the Hippo pathway, Merlin functions as solid-like condensates to activate Hippo signaling. For purposes of clarity, we shall use “Merlin” as a non-species-specific reference to this evolutionarily conserved protein; “Mer” and “NF2” will be used to refer to the corresponding *Drosophila* and mammalian orthologue, respectively.

## Merlin forms solid-like condensates *in vivo*

To investigate the physiological regulation of Mer *in vivo*, we fused mNeonGreen to the C-terminus of Mer (Mer-mNG) and expressed the fusion protein under the ubiquitous *tubulin* promoter in *Drosophila*. Mer-mNG was expressed in amounts comparable to those of endogenous Mer (fig. S1A) and rescued the lethality caused by a *mer* loss-of-function mutation (see Material and Methods). Mer-mNG formed distinct puncta in the cell cortex in ovarian follicle cells and nurse cells (fig. S1, B and C). A similar pattern was observed for endogenous Mer or hemagglutinin (HA)-tagged Mer (Mer-HA) (fig. S1C). The formation of Mer puncta in nurse cells appeared to be developmentally regulated, because the intensity of the punctate Mer signal increased and the intensity of the surrounding non-punctate Mer signal decreased as the egg chamber developed from stage 9 to stage 11 (fig. S1D). The punctate appearance of Mer-mNG is reminiscent of biomolecular condensates formed through liquid-liquid phase separation (LLPS), which often adopt a spherical shape due to surface tension (3). Because the small, diffraction-limited size of Mer puncta in ovarian nurse cells made it impossible to conduct shape analysis by confocal microscopy, we analyzed Mer-mNG expressed in *Drosophila* embryo-derived S2R+ cell line. Mer formed distinct puncta with variable sizes but always with spherical shapes (an aspect ratio of 1.18) in S2R+ cells (fig. S1, E and F), indicating a possible origin in LLPS. However, unlike liquid-like condensates for which two touching condensates often fuse and relax into one condensate with increased size (1–5), we never observed fusion between two contacting Mer-mNG puncta in nurse cells, arguing against a liquid-like property (fig. S1G).

**Fig. 1.**
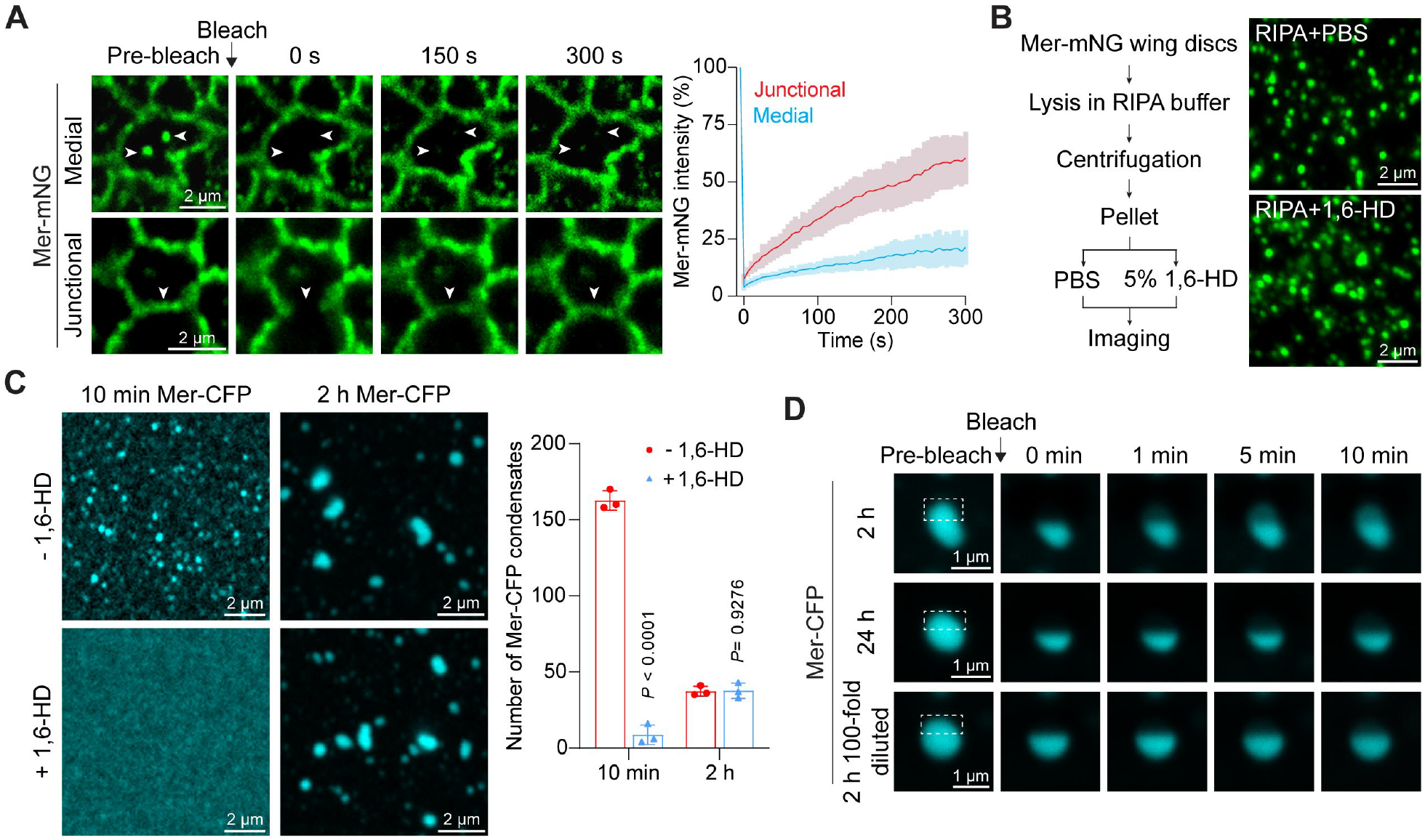
Mer forms solid-like condensates *in vivo* and *in vitro*. (**A**) FRAP analysis of medial apical and junctional Mer-mNG in wing discs. Arrowheads mark the photobleached region. Quantification of fluorescence intensity is shown to the right. Data are represented as mean ± s.d., *n* = 13 (medial), *n* = 21 (junctional). (**B**) Wing discs expressing Mer-mNG were lysed in RIPA lysis buffer (50 mM Tris-HCl pH 7.4, 150 mM NaCl, 1% Nonidet P-40, 0.5% sodium deoxycholate, 0.1% SDS) followed by centrifugation. Pellets were treated with PBS or 5% 1,6-hexanediol (1,6-HD) before imaging. Note that Mer-mNG puncta were not dissolved by detergents and 1,6-HD. (**C**) *in vitro* reconstituted Mer-CFP condensates at indicated time points, without or with 5% 1,6-HD treatment. Quantification of the number of Mer-CFP condensates is shown to the right. Data are represented as mean ± s.d., *n* = 3 for each measurement, unpaired two-tailed Student’s *t*-test. (**D**) Partial-FRAP analysis (only half of an individual *in vitro* reconstituted Mer-CFP condensate was photobleached, marked by a dashed box) of 2-hour-old, 24-hour-old condensates, or 2-hour-old condensates after the entire reaction was diluted by 100-fold.

We characterized the subcellular localization of Mer in the developing epithelia of wing imaginal discs (fig. S1H). Similar to endogenous Mer, Mer-mNG was detected as both medial apical puncta and a continuous belt on the cell junction (fig. S1I). We analyzed the dynamics of the two subcellular pools of Mer protein in wing disc cells using fluorescent recovery after photobleaching (FRAP). The fluorescence recovery of medial apical Mer puncta was much slower than that of junctional Mer after photobleaching (Fig. 1A). Consistent with their less dynamic nature, the medial apical Mer puncta were resistant to solubilization by lysis buffer containing both nonionic and ionic detergents (RIPA buffer) (Fig. 1B). The Mer puncta that survived lysis by RIPA buffer were further resistant to solubilization by 1,6-hexanediol, a chemical that dissolves liquid-like but not solid-like condensates (22) (Fig. 1B). Medial apical Mer, but not the junctional Mer, preferentially mediates Mer function in Hippo pathway activation (23). Thus, different subcellular pools of Mer in wing disc cells may exist in distinct physical states, with the medial apical Mer puncta in a functional and less dynamic state, and the junctional Mer in a less functional and more dynamic state. In comparison, liquid-like condensates formed by another Hippo pathway upstream regulator Kibra (16, 24) were much more dynamic (fig. S2A). Consistent with these findings, when expressed in *Drosophila* S2R+ cells, Mer, but not Kibra, was resistant to solubilization by detergent (fig. S2B).

Similar to its *Drosophila* counterpart, the human NF2 protein also formed spherical condensates (fig. S2C), and was resistant to solubilization by detergent (fig. S2D). Together, these results suggest that Merlin forms biomolecular condensates with solid-like material properties.

## Merlin forms solid-like condensates *in vitro* that age with time

To understand how Mer forms solid-like condensates, we purified bacterially expressed Mer-mTurquoise2 (Mer-CFP) recombinant protein at high salt concentration and then reduced salt concentration to physiological level to induce condensate formation. Small Mer-CFP condensates (~0.2 μm diameter) started to appear at 10 min (fig. S3, A and B). The Mer-CFP condensates grew in size and reached a maximal size (1 to 2 μm diameter) in 2 hours (fig. S3A). Unresolved fusion intermediates or condensates sticking together, signs of decreased protein dynamics and increased solid-like material properties (3, 10, 25–27), were often seen starting from 1 hour after the induction of condensate formation (fig. S3A). Consistent with these results, cryo-electron tomography (Cryo-ET) revealed smaller size and spherical shape of 10-minute-old condensates, and larger size and unresolved fusion intermediates of 2-hour-old condensates (fig. S3C). Whereas both young and old condensates were amorphous in morphology, 2-hour-old condensates had higher internal protein density (fig. S3C), consistent with the latter being in a more solid-like state. Consistent with the aging process, 10-minute-old condensates were completely dissolved by 1,6-hexanediol whereas 2-hour-old condensates were stable under the same condition (Fig. 1C).

Further supporting the aging process, partial-FRAP analysis (only half of an individual Mer-CFP condensate was photobleached) revealed fluorescence recovery of the photobleached area in 2-hour-old, but not 24-hour-old condensates (Fig. 1D). In principle, the recovery of fluorescence signal in partial-FRAP analysis of 2-hour-old condensates may result from the incorporation of Mer-CFP from the dilute phase into the condensed phase, or alternatively, internal diffusion of Mer-CFP within the same condensate from the unbleached to the bleached area. To distinguish between these possibilities, we induced condensate formation for 2 hours and then diluted the entire reaction 100-fold (which in effect reduced Mer-CFP concentration in the dilute phase by 100-fold) before partial-FRAP analysis. Under this condition, the fluorescent signal remained stable in both photobleached and un-photobleached areas, suggesting no diffusion within the condensate or exchange between the condensed and the dilute phases (Fig. 1D). Thus, although both appeared as solid-like condensates, the 2-hour-old condensates could incorporate Mer protein from the dilute phase whereas the 24-hour-old condensates were completely static, reflecting the aging of *in vitro* reconstituted Mer condensates.

Like its *Drosophila* counterpart, purified NF2-monomeric enhanced green fluorescent protein (NF2-mEGFP) also formed spherical condensates that grew in size to 1 to 2 μm in diameter by 2 hours after the induction of condensate formation (fig. S3, B and D). NF2-mEGFP condensates frequently fused with each other after 2 hours, eventually forming fiber-like structures (fig. S3D). Although 10-minute-old condensates were completely dissolved by 1,6-hexanediol, the aged, fiber-like NF2-mEGFP condensates were resistant to 1,6-hexanediol (fig. S3E). Thus, purified Merlin protein forms solid-like condensates that age with time.

## Coiled-coil domain-mediated oligomerization is required for Mer condensation and plasma membrane association

The intrinsic ability of purified Mer protein to form condensates indicates that Mer may self-associate into higher-order assemblies. Supporting this view, chemical crosslinking revealed that Mer readily formed high-order oligomers in S2R+ cells (fig. S4A). Mer contains an N-terminal 4.1 protein, ezrin, radixin, moesin (FERM) domain, a central coiled-coil domain (CC), and a C-terminal tail (CTT) domain (fig. S4B). To investigate which domain of Mer mediates oligomerization, we conducted similar crosslinking analysis on individual domains of Mer expressed in S2R+ cells. The coiled-coil domain was sufficient for oligomerization (fig. S4C). Interactions between coiled-coil α-helices are mediated by hydrophobic residues at the *a* and *d* positions of its multiple heptad repeats (fig. S4D) (28). Mutating six hydrophobic residues at these positions in the CC domain to proline (mutCC) diminished not only the oligomerization of the CC domain, but also the ability of purified Mer-CFP protein to form condensates *in vitro* (fig. S4, E to G).

**Fig. 2.**
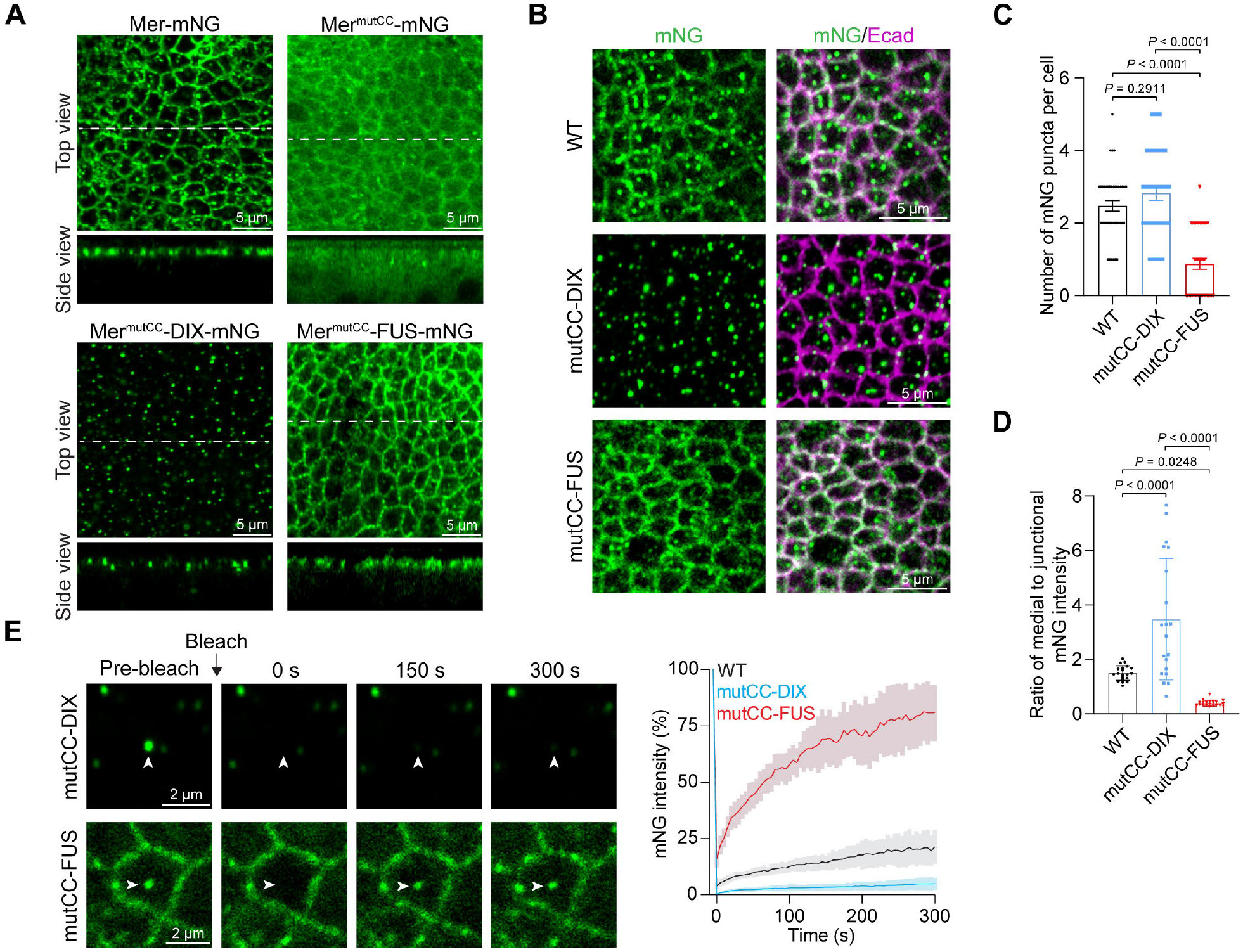
Solid-like material properties of Mer condensates dictate its medial apical localization. (**A**) Wing discs expressing Mer-mNG (WT), Mer^mutCC^-mNG (mutCC), Mer^mutCC^-FUS-mNG (mutCC-DIX) or Mer^mutCC^-DIX-mNG (mutCC-FUS). Side views along the dashed lines are shown below. (**B**) Wing discs expressing Mer-mNG, Mer^mutCC^-DIX-mNG or Mer^mutCC^-FUS-mNG were stained for Ecad (*Drosophila* E-Cadherin labeling cell junctions). (**C**) Quantification of the number of medial apical puncta per cell in (B). Note that Mer^mutCC^-FUS formed fewer condensates than Mer and Mer^mutCC^-DIX. Data are represented as mean ± s.e.m., *n* = 40 cells from 4 discs for each protein, one-way ANOVA with Tukey’s test. (**D**) Quantification of the ratio of medial apical mNG intensity to junctional mNG intensity per cell in (B). Note that, unlike Mer and Mer^mutCC^-DIX, Mer^mutCC^-FUS showed higher junctional intensity than medial intensity (a mean medial to junctional intensity ratio of 0.39). Data are represented as mean ± s.d., *n* = 20 for each protein, one-way ANOVA with Tukey’s test. (**E**) FRAP analysis of medial Mer^mutCC^-DIX-mNG and Mer^mutCC^-FUS-mNG condensates in wing discs (marked by arrowheads). Quantification of fluorescence intensity is shown to the right. Data are represented as mean ± s.d., *n* = 20 (DIX), *n* = 21 (FUS). FRAP analysis of wild-type (WT) Mer-mNG condensates (Fig. 1A) is also included for comparison.

Consistent with *in vitro* characterization, unlike Mer-mNG, Mer^mutCC^-mNG did not form medial apical puncta in wing imaginal discs (Fig. 2A). Besides loss of the medial apical pool, the normal cell junction pool of Mer was also greatly diminished by the Mer^mutCC^ mutant (Fig. 2A). Instead, Mer^mutCC^-mNG was diffusely localized in the cytoplasm (Fig. 2A). Similar results were seen in S2R+ cells, in which Mer-mNG was detected as spherical shaped puncta on the plasma membrane, whereas Mer^mutCC^-mNG was diffusely localized in the cytoplasm (fig. S5A). Thus, Mer condensation appears to enhance its membrane association. To further test this model, we sought to restore Mer condensation by fusing the low-complexity domain of the RNA binding protein Fused In Sarcoma (FUS), which is commonly used to restore LLPS (10, 29, 30), or the dishevelled and axin (DIX) domain of human Dishevelled (Dvl), which polymerizes through head-to-tail association (31–33), to the C-terminus of Mer^mutCC^ (fig. S5B). Both Mer^mutCC^-FUS and Mer^mutCC^-DIX restored condensate formation as well as plasma membrane association in S2R+ cells (fig. S5A). Thus, we conclude that CC-mediated oligomerization is required for both solid-like condensation and plasma membrane association of Mer.

## The solid-like material properties of Mer condensates dictate its medial apical localization

Despite their similar ability to form plasma membrane-associated condensates in S2R+ cells as shown above, FRAP analysis revealed distinct protein dynamics in condensates formed by wild-type Mer, Mer^mutCC^-DIX and Mer^mutCC^-FUS. Although wild-type and Mer^mutCC^-DIX condensates were non-dynamic, Mer^mutCC^-FUS condensates were highly dynamic (fig. S5C). Time-lapse imaging revealed that upon contact, Mer^mutCC^-DIX condensates stuck with each other but did not fuse whereas Mer^mutCC^-FUS condensates rapidly fused with each other and relaxed into a single spherical condensate (fig. S5D), indicating that the former are solid-like and the latter are liquid-like.

As in S2R+ cells, Mer^mutCC^-DIX and Mer^mutCC^-FUS also restored the plasma membrane association in wing imaginal discs (Fig. 2A). Despite that, their relative subcellular distribution between the medial apical cortex and cell junctions was very different. Unlike wild-type Mer, which was localized to both medial apical condensates and a continuous belt on the cell junction in wing imaginal disc cells (Fig. 2, B to D), Mer^mutCC^-DIX was detected only as condensates with 90% of the condensates localized to the medial apical cortex and 10% of the condensates localized to the junctions (Fig. 2, B to D), whereas Mer^mutCC^-FUS was predominantly detected as a continuous belt on the cell junction with fewer condensates at medial apical cortex (Fig. 2, B to D). FRAP analysis revealed that the Mer^mutCC^-DIX condensates were less dynamic than were the wild-type Mer condensates, whereas the Mer^mutCC^-FUS condensates were much more dynamic than were the wild-type Mer condensates (Fig. 2E). Such differences appeared not to be due to protein abundance because Mer^mutCC^-DIX and Mer^mutCC^-FUS were expressed in similar amounts (fig. S5E). Thus, the material properties of Mer condensates appear to affect their subcellular localization, with the solid-like Mer^mutCC^-DIX having medial apical localization and the liquid-like Mer^mutCC^-FUS having junctional localization.

## Solid-like Mer condensates regulate the organization and signaling output of the Hippo kinase cascade

**Fig. 3.**
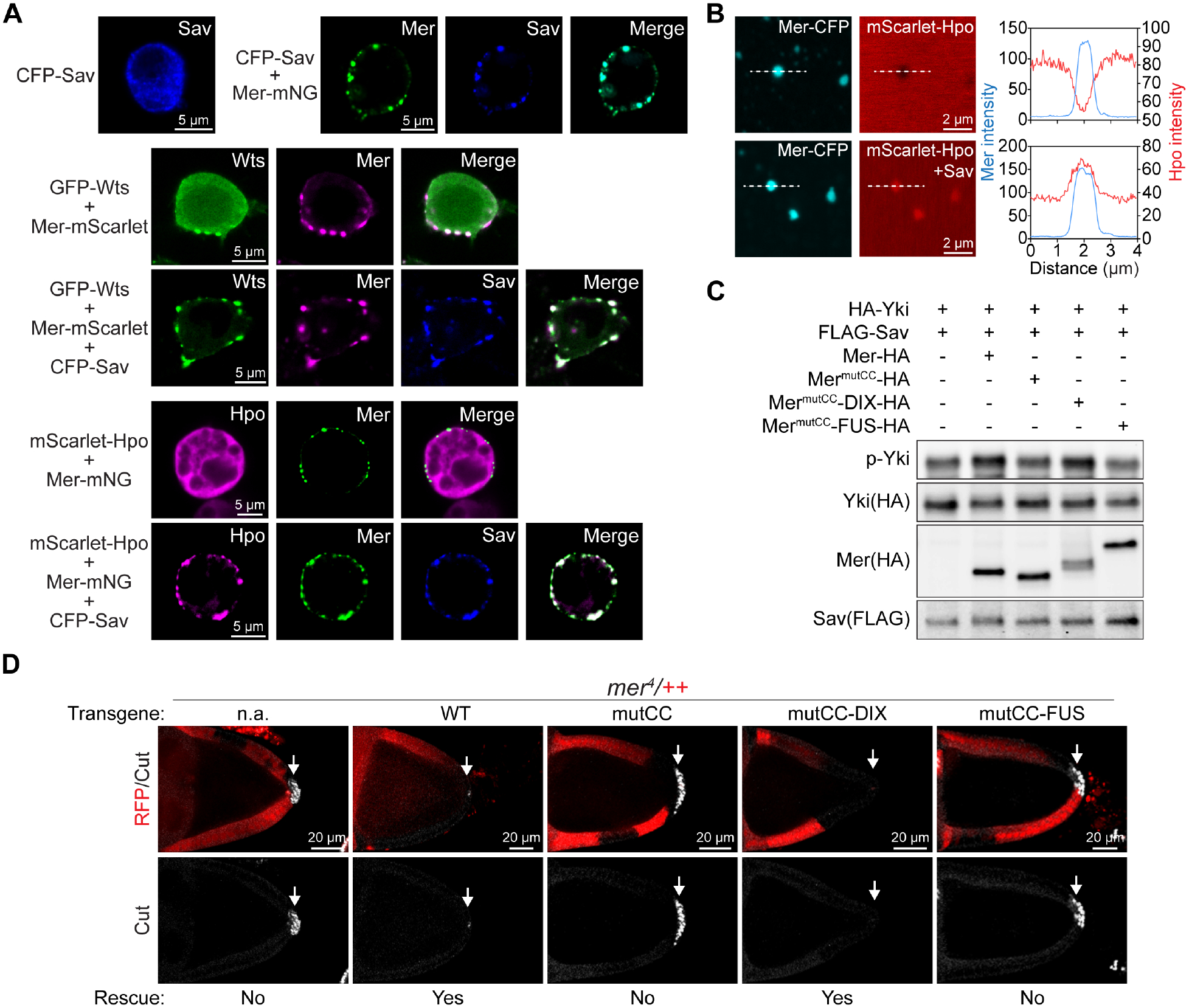
Solid-like Mer condensates regulate the organization and signaling output of the Hippo kinase cascade. (**A**) S2R+ cells expressing the indicated proteins showing that the membrane-associated Mer condensates enriched Hippo pathway components. (**B**) *in vitro* phase separation assay showing that Mer-CFP condensates enriched Hpo-mScarlet in the presence of Sav. Quantifications of fluorescence intensity along the dashed lines are shown to the right. (**C**) S2R+ cells expressing the indicated proteins were analyzed by western blot for Yki phosphorylation. Note that wild-type Mer and Mer^mutCC^-DIX but not Mer^mutCC^ and Mer^mutCC^-FUS enhanced Yki phosphorylation. (**D**) Follicle cells in stage 10 egg chamber containing RFP-negative *mer*^*4*^ clones and expressing Mer-mNG or the indicated variants were stained for Cut. Note that only wild-type Mer-mNG and Mer^mutCC^-DIX-mNG rescued the ectopic Cut expression in posterior *mer*^*4*^ mutant follicle cells.

We investigated how Mer condensation mediates its activity in Hippo signaling. Consistent with direct Mer-Wts and Mer-Sav protein interactions (20, 34), the membrane localized Mer condensates in S2R+ cells contained Wts and Sav (Fig. 3A). Association of Wts with Mer condensates was enhanced in cells also expressing Sav (Fig. 3A). Even though Mer does not directly bind Hpo (20), the Mer condensates contained Hpo in the presence of Sav, both in S2R+ cells and in *in vitro* reconstitutions (Fig. 3, A and B). Thus, the Mer condensates appear to spatially organize the core kinase cascade of the Hippo pathway.

To further assess the function of Mer condensation in Hippo signaling, we analyzed phosphorylation of the Yorkie (Yki) protein as an indicator of pathway activity. Wild-type Mer, but not Mer^mutCC^, stimulated Yki phosphorylation in S2R+ cells, indicating that Mer condensation is important for its activity in Hippo signaling (Fig. 3C). Furthermore, the liquid-like Mer^mutCC^-FUS did not stimulate Yki phosphorylation but the solid-like Mer^mutCC^-DIX did (Fig. 3C). Thus, the solid-like material properties of Mer condensates appear to be important for Mer function in Hippo signaling.

Loss-of-*mer* results in ectopic expression of *cut* (a gene required for proper differentiation of *Drosophila* follicle cells) in posterior follicle cells (PFCs) in stage 10 egg chambers, a hallmark of defective Hippo signaling in oogenesis (35, 36). This mutant phenotype provides a convenient assay to compare the *in vivo* activity of the various Mer mutants described above. Wild-type Mer, but not the condensation-defective Mer^mutCC^, restored *mer* function in loss-of-*mer* mutants (Fig. 3D). Although Mer^mutCC^-DIX, which forms solid-like condensates localized to the medial apical cortex, also restored *mer* function (Fig. 3D), the more junctional localized and liquid-like Mer^mutCC^-FUS did not (Fig. 3D). Thus, the solid-like material properties of Mer condensates appear to dictate their localization and function *in vivo*. These findings further implicate medial apical but not junctional Mer as the active subcellular pool in Hippo pathway activation in *Drosophila* epithelia.

## Plasma membrane association of Mer requires both FERM domain-mediated PI4P-binding and CC domain-mediated oligomerization

The plasma membrane association of NF2 and related ERM proteins such as Radixin is mediated by direct binding between specific positively charged lipid-binding residues in the FERM domain and negatively charged phosphatidylinositol 4,5-bisphosphate (PIP2) on the plasma membrane through electrostatic interactions (37–39). Given our current study showing that CC domain-mediated oligomerization promotes Mer plasma membrane association, the simplest hypothesis is that normal plasma membrane association of Mer and NF2 requires both FERM domain-mediated binding of phosphoinositides and CC domain-mediated oligomerization. We conducted additional experiments to further test this model.

Because studies implicating FERM-PIP2 binding in plasma membrane association of NF2 were done in cultured mammalian cells, we confirmed whether the same is true for *Drosophila* Mer. *Drosophila* also provided an opportunity to assess the importance of FERM-PIP2 binding in intact epithelial tissues. Mutating critical residues implicated in direct phosphoinositide binding in the FERM domain (37, 38) (K67, K70 and K288 to alanine, Mer^3A^) abolished membrane association of Mer in ovarian follicle cells and S2R+ cells. Mer^3A^ was diffusely localized throughout the cytoplasm and did not form distinct condensates (fig. S6, A and B).

We tested whether PIP2 is required for plasma membrane association of Mer by examining mutant clones lacking critical enzymes for plasma membrane PIP2 synthesis. As a control, we also included enzymes critical for synthesizing plasma membrane phosphatidylinositol 4-phosphate (PI4P), the second most abundant phosphoinositide on the plasma membrane. We first examined mutant clones of *skittles* (*sktl*), which encodes the major phosphatidylinositol-4-phosphate 5-kinase (PI4P5K) responsible for the generation of PIP2 on the plasma membrane (40), and whose inactivation greatly diminished membrane PIP2 but had little effect on PI4P level in wing imaginal discs, as revealed by the PIP2 sensor GFP-PLCδ1-PH (41) and the PI4P sensor GFP-2xOsh2-PH (fig. S6, C to E). Unexpectedly, despite decreased PIP2 level, plasma membrane association of endogenous Mer was unaffected in *sktl* mutant cells (fig. S6, D and E).

**Fig. 4.**
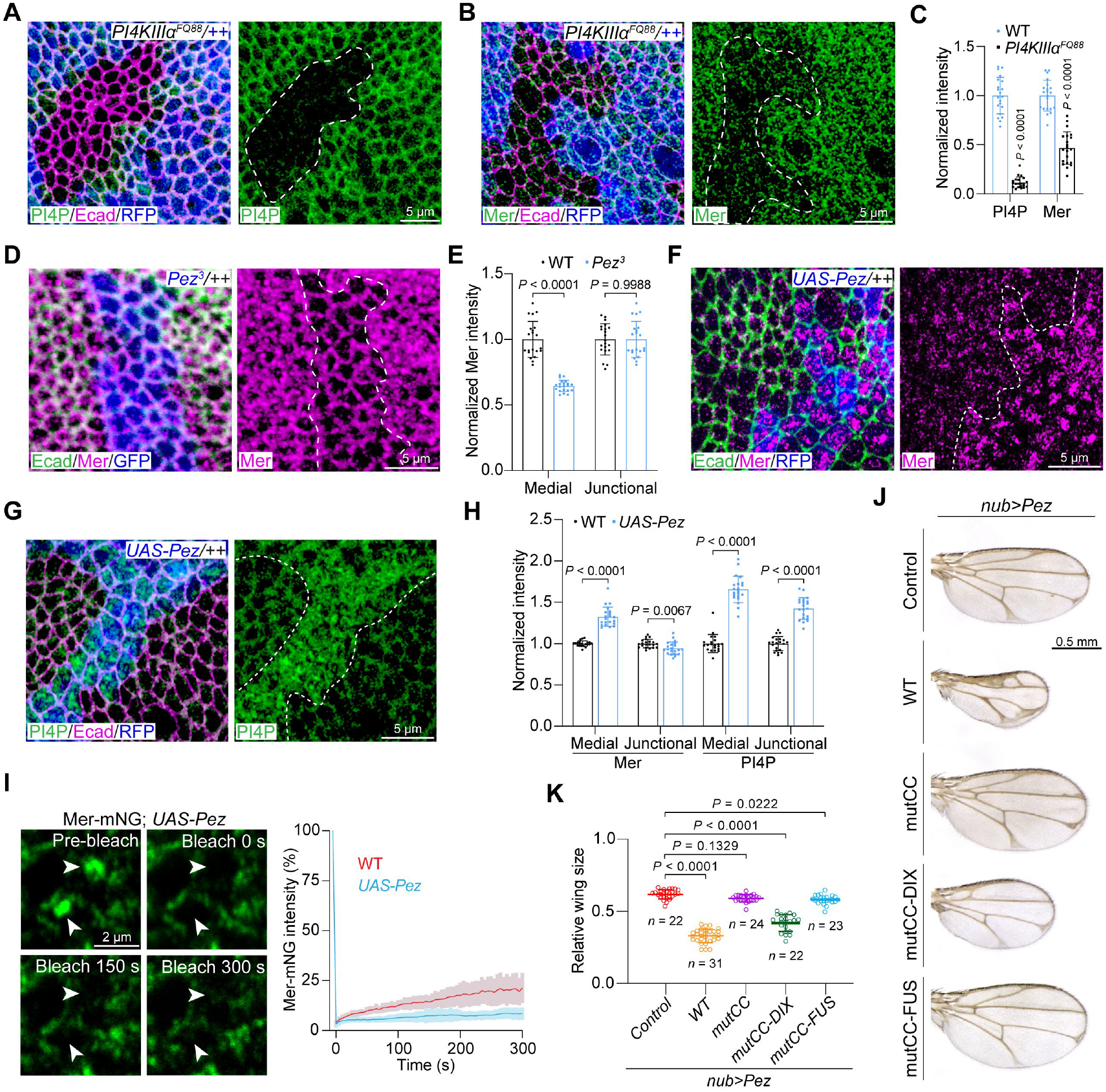
Pez promotes medial apical condensation of Mer by increasing plasma membrane PI4P level. (**A** and **B**) Wing discs containing RFP-negative clones of *PI4KIIIα*^*FQ88*^. PI4P was labeled by expression of GFP-2xOsh2-PH (A) and Mer was labeled by immunostaining (B). (**C**) Quantification of GFP-2xOsh2-PH and Mer fluorescence intensity in (A and B) showing reduced PI4P and Mer in *PI4KIIIα*^*FQ88*^ mutant clones. Data are represented as mean ± s.d., *n* = 21, unpaired two-tailed Student’s *t*-test. (**D**) A wing disc containing GFP-positive *Pez*^*3*^ clones was stained for Mer and Ecad. (**E**) Quantification of medial apical and junctional Mer fluorescence intensity in (D) showing reduced medial but not junctional Mer in *Pez*^*3*^ mutant clones. Data are represented as mean ± s.d., *n* = 21, unpaired two-tailed Student’s *t*-test. (**F** and **G**) Wing discs containing RFP-positive Pez-overexpressing clones were stained for Ecad. Mer was labeled by immunostaining (F) and PI4P was labeled by expression of GFP-2xOsh2-PH (G). (**H**) Quantification of medial and junctional Mer or GFP-2xOsh2-PH fluorescence intensity in (F and G) showing the increase of both Mer and PI4P in Pez-expressing clones. Data are represented as mean ± s.d., *n* = 21, unpaired two-tailed Student’s *t*-test. (**I**) FRAP analysis of Mer-mNG condensates in Pez-expressing wing discs. Arrowheads mark the two photobleached regions. Quantification of fluorescence intensity is shown to the right, *n* = 20. FRAP analysis of medial Mer-mNG condensates in wild-type wing discs (Fig. 1A) is also included for comparison. (**J**) Adult wings of *nub*-*Gal4;UAS*-*Pez* flies carrying wild-type Mer or the indicated variants under the control of the tubulin promoter. (**K**) Quantification of wing size in (J). Data are represented as mean ± s.d., one-way ANOVA with Tukey’s test. Note that none of the tubulin-driven Mer transgenes caused visible wing phenotype by itself (see fig. S10, F and G).

We examined mutant clones of *PI4KIIIα*, which encodes the major enzyme that synthesizes and maintains the plasma membrane PI4P (42), and whose inactivation depleted PI4P but had little effect on PIP2 on plasma membrane in wing imaginal discs (43) or ovarian follicle epithelial cells (Fig. 4, A and C, and fig. S6, F to I). Accompanying the decreased PI4P level, plasma membrane association of endogenous Mer was diminished in *PI4KIIIα* mutant cells in both tissues (Fig. 4, B and C, and fig. S6J). This reduction appeared not be due to compromised transcription of endogenous *mer* gene, because Mer-HA expressed from a heterologous promoter was also mis-localized throughout the cytoplasm in *PI4KIIIα* mutant cells (fig. S6K). Thus, in *Drosophila* epithelia, the plasma membrane association of Mer is selectively dependent on PI4P but not PIP2.

We tested whether Mer can directly bind PI4P. When expressed with PI4P or PIP2 sensor in S2R+ cells, Mer-mScarlet localized with PI4P-containing but not PIP2-containing microdomains on the plasma membrane (fig. S7A). In protein-lipid overlay assay, purified Mer-CFP bound to PI4P, but not PIP2 or PIP3 (fig. S7B). Because FERM-PI4P binding appears to directly mediate Mer membrane association, we explored how CC domain-mediated oligomerization might promote such association. In our protein-lipid overlay assay, the oligomerization-defective Mer^mutCC^ mutant exhibited an overall reduction (by 4.3-fold) of PI4P-binding without losing its selectivity towards PI4P relative to the other phosphoinositides (fig. S7B). Restoring condensation by Mer^mutCC^-DIX or Mer^mutCC^-FUS also restored PI4P binding, as revealed by co-localization with PI4P-positive microdomains in S2R+ cells (fig. S7C). Thus, although the FERM domain mediates direct PI4P binding, CC domain-mediated oligomerization nonetheless impacts PI4P-binding and thus Mer plasma membrane association, likely by increasing the overall valency of Mer-PI4P binding.

## Pez promotes medial apical condensation of Mer by increasing plasma membrane PI4P level

After uncovering the importance of PI4P in plasma membrane association of Mer, we sought to identify PI4P-dependent regulators of Mer. Because Mer and another FERM domain protein Expanded (Ex) represent two parallel modules that function additively to regulate the core Hippo kinase cascade (17), we reasoned that genetic enhancers of the *ex* loss-of-function mutant phenotype might uncover genes acting in the Mer module. By performing chemical mutagenesis screens for mutations that enhance the overgrowth phenotype of *ex* mutants, we isolated a nonsense mutation causing early truncation (L128 to stop) in *Pez* (*Pez*^*3*^) that enhanced the *ex*^*e1*^ eye overgrowth phenotype, even though the *Pez*^*3*^ mutation by itself did not cause visible eye overgrowth (fig. S8, A and B).

Pez and its mammalian counterpart Protein Tyrosine Phosphatase Non-Receptor Type 14 (PTPN14) are positive regulators of Hippo signaling, albeit through poorly understood mechanisms (44, 45). Our identification of a *Pez* mutation as an enhancer of loss-of-*ex* mutant phenotype indicates that Pez functions in parallel with Ex, and likely in the Mer module. Further experiments supported this model. The small wing phenotype resulting from *Pez* overexpression (44) by the *nub*-Gal4 driver (*nub>Pez*) was specifically potentiated by an additional dosage of *mer* expressed from a *tubulin* promoter (*tubulin*-*mer* was otherwise non-consequential by itself), but not by a similar dosage of *ex* (fig. S8, C and D). Also, the small-wing phenotype of *nub>Pez* animals was suppressed in wings lacking *mer* but not *ex*, placing *Pez* genetically upstream of *mer* (fig. S8, C and D).

We investigated how Pez acts upstream of Mer. In ovarian follicle cells, the apical membrane association of endogenous Mer was reduced in *Pez* mutant cells (fig. S8E). This reduction appeared not to be due to reduced *mer* gene expression because Mer-HA expressed from a heterologous promoter also lost its apical association in *Pez* mutant cells (fig. S8E). Similarly, in the wing imaginal discs, loss-of-*Pez* resulted in a depletion of medial apical Mer but not junctional Mer (Fig. 4, D and E), whereas overexpression of *Pez* promoted the formation of medial apical Mer puncta and a mild decrease in abundance of junctional Mer (Fig. 4, F and H). Consistent with the role of medial apical Mer in organizing Hippo kinase cascade and Yki phosphorylation (Fig. 3, A to C), Pez overexpression not only induced the medial apical accumulation of Hpo, Sav and Wts but also decreased the expression of the Yki target gene *expanded* (*ex*). Both of these effects were diminished in cells lacking *mer* (fig. S9). Like its *Drosophila* counterpart, PTPN14 also induced accumulation of medial apical but not junctional NF2 when overexpressed in Madin-Darby canine kidney (MDCK) cells (fig. S8F). Together, these results implicate a role for Pez and PTPN14 in Hippo pathway activation by promoting the medial apical cortex localization of Mer and NF2, respectively.

Because membrane association of Mer requires PI4P-binding, we tested whether Pez regulates PI4P on the plasma membrane. Indeed, the abundance of PI4P was significantly reduced in *Pez* mutant cells compared to adjacent wild-type cells in mosaic wing discs (fig. S10, A and C). Conversely, *Pez* overexpression increased abundance of PI4P at both medial apical cortex and cell junctions with a net increase in the relative ratio of medial apical over junctional concentration of PI4P. Supporting PI4P as a mediator between Pez and Mer localization, knockdown or knockout of *PI4KIIIα* in *Pez*-overexpressing cells diminished both abundance of PI4P and membrane association of Mer (fig. S10, B to E). In contrast, the medial apical accumulation of Mer induced by Pez overexpression was not accompanied by accumulation of Kibra and was genetically independent of *kibra* (fig. S10, F and G). Together, these data indicate that Pez promotes medial apical enrichment of Mer by increasing production of PI4P.

Because Pez promotes the medial apical enrichment of Mer, we examined how such enrichment affects Mer condensation. FRAP analysis revealed that the low protein dynamics of medial apical Mer condensates was further decreased by Pez overexpression (Fig. 4I). Thus, Pez-mediated increases in PI4P appears to not only promote medial apical localization of Mer but also to drive the condensates to a more solid-like state. Consistent with Pez’s role in promoting solid-like condensation of Mer, wild-type Mer and Mer^mutCC^-DIX, both of which were capable of forming solid-like condensates, but not the condensation-defective Mer^mutCC^ or the liquid-like Mer^mutCC^-FUS, enhanced the small-wing phenotype of *nub>pez* animals (Fig. 4, J and K, and fig. S10, H and I).

## Cytoskeletal tension suppresses medial apical Mer condensates but increases junctional diffusive Mer

Given that Pez positively regulates Hippo signaling by promoting the formation of medial apical Mer condensates, we considered whether negative regulators of Hippo signaling might suppress the Hippo pathway by decreasing the medial apical Mer condensates.

**Fig. 5.**
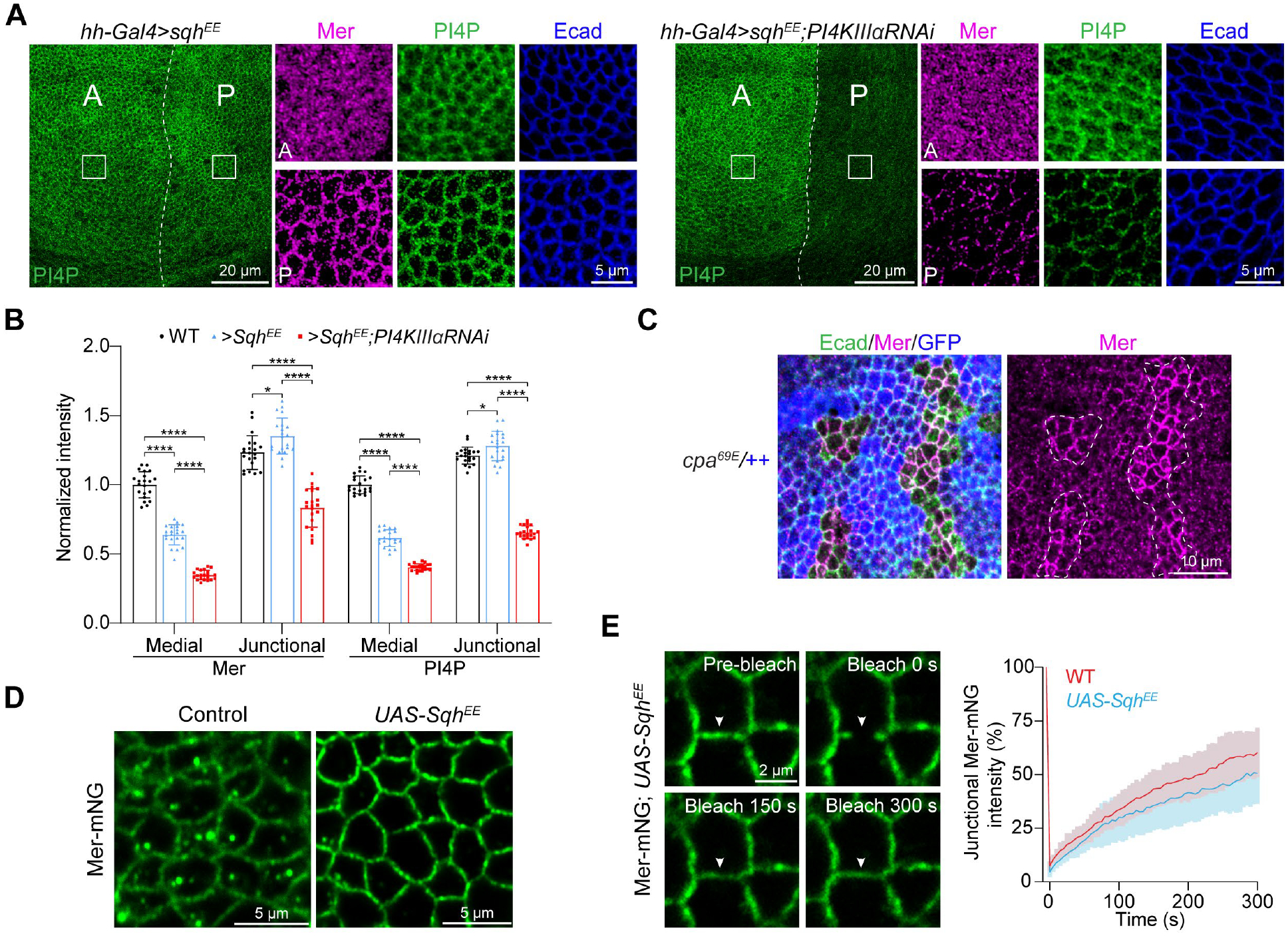
Cytoskeletal tension suppresses medial apical Mer and PI4P while increasing junctional Mer and PI4P. (**A**) Wing discs overexpressing Sqh^EE^ or Sqh^EE^ with *PI4KIIIα* RNAi in the posterior compartment using the *hh*-*Gal4* driver. PI4P was labeled by expression of GFP-2xOsh2-PH and Mer was labeled by immunostaining. The boxed area in the anterior (A) and posterior (P) compartments are shown to the right at higher magnification. (**B**) Quantification of medial apical and junctional GFP-2xOsh2-PH and Mer fluorescence intensity in (A). Note that overexpression of Sqh^EE^ reduced medial apical but increased junctional Mer and PI4P, and *PI4KIIIα* RNAi diminished both pools of Mer/PI4P. Data are represented as mean ± s.d., *n* = 21, one-way ANOVA with Tukey’s test, *P < 0.05, ****P < 0.0001. (**C**) A wing disc containing GFP-negative *cpa*^*69E*^ clones were stained for Ecad and Mer. Note the increase of junctional Mer in *cpa*^*69E*^ mutant cells. (**D**) Mer-mNG wing discs without or with Sqh^EE^ overexpression. Note that Sqh^EE^ suppressed medial apical Mer condensates but increased junctional diffusive Mer. (**E**) FRAP analysis of junctional Mer-mNG in Sqh^EE^-overexpressing wing disc cells. Arrowheads mark the photobleached region. Quantification of fluorescence intensity is shown to the right. Data are represented as mean ± s.d., *n* = 18. FRAP analysis of junctional Mer-mNG in wild-type wing disc cells (Fig. 1A) is also included for comparison.

Because cytoskeletal tension is one of the best characterized negative regulators of Hippo signaling, we tested whether increased cell tension affected the subcellular localization of Mer. We used two genetic manipulations that increase cytoskeletal tension in wing imaginal disc cells: expressing a constitutively active myosin light chain variant (Sqh^EE^) or knocking out the F-actin-capping protein Cpa (46–48). Both conditions resulted in an increase of endogenous Mer at the cell junction accompanied by a decrease of medial apical Mer (Fig. 5, A to C).

We noticed that in Sqh^EE^-overexpressing cells, the junctional Mer maintained the appearance of a continuous belt at the cell junction as in wild-type cells (Fig. 5D). This indicates that despite the increased Mer concentration at the cell junction in Sqh^EE^-overexpressing cells, the junctional Mer remained in a dynamic or diffusive state as in wild-type cells, which was confirmed by FRAP analysis (Fig. 5E).

The differential regulation of the two subcellular Mer pools by cytoskeletal tension was accompanied by corresponding changes in concentration of PI4P, as Sqh^EE^-overexpressing cells also showed an increased abundance of junctional PI4P and a decrease of medial apical PI4P (Fig. 5, A and B). In cells overexpressing Sqh^EE^ but lacking *PI4KIIIα*, both pools of Mer and PI4P were depleted (Fig. 5, A and B), consistent with PI4P being a critical determinant of Mer plasma membrane association. These results indicate that cytoskeletal tension differentially regulates the relative distribution of PI4P and Mer between the medial apical cortex and the cell junction. Together with our analysis of Pez, we conclude that Pez and cytoskeletal tension antagonistically regulate the physical states and relative distribution of distinct subcellular Mer pools between the medial apical cortex and the cell junction.

## Cytoskeletal tension promotes a physical state transition and disassembly of Mer condensates

To gain further insights into how cytoskeletal tension suppresses Mer condensates, we changed osmolarity as an acute way to modulate cytoskeletal tension (49, 50). Consistent with the results from stable genetic increase of cytoskeletal tension, acute increases of cytoskeletal tension by hypoosmotic stress also suppressed abundance of medial apical Mer condensates and PI4P while increasing junctional accumulation of diffusive Mer and PI4P (fig. S11, A and B). Conversely, hyperosmotic conditions (corresponding to lower-than-normal tension) promoted medial apical accumulation of Mer condensates and PI4P accompanied by a loss of junctional Mer and PI4P (fig. S11, A to C).

**Fig. 6.**
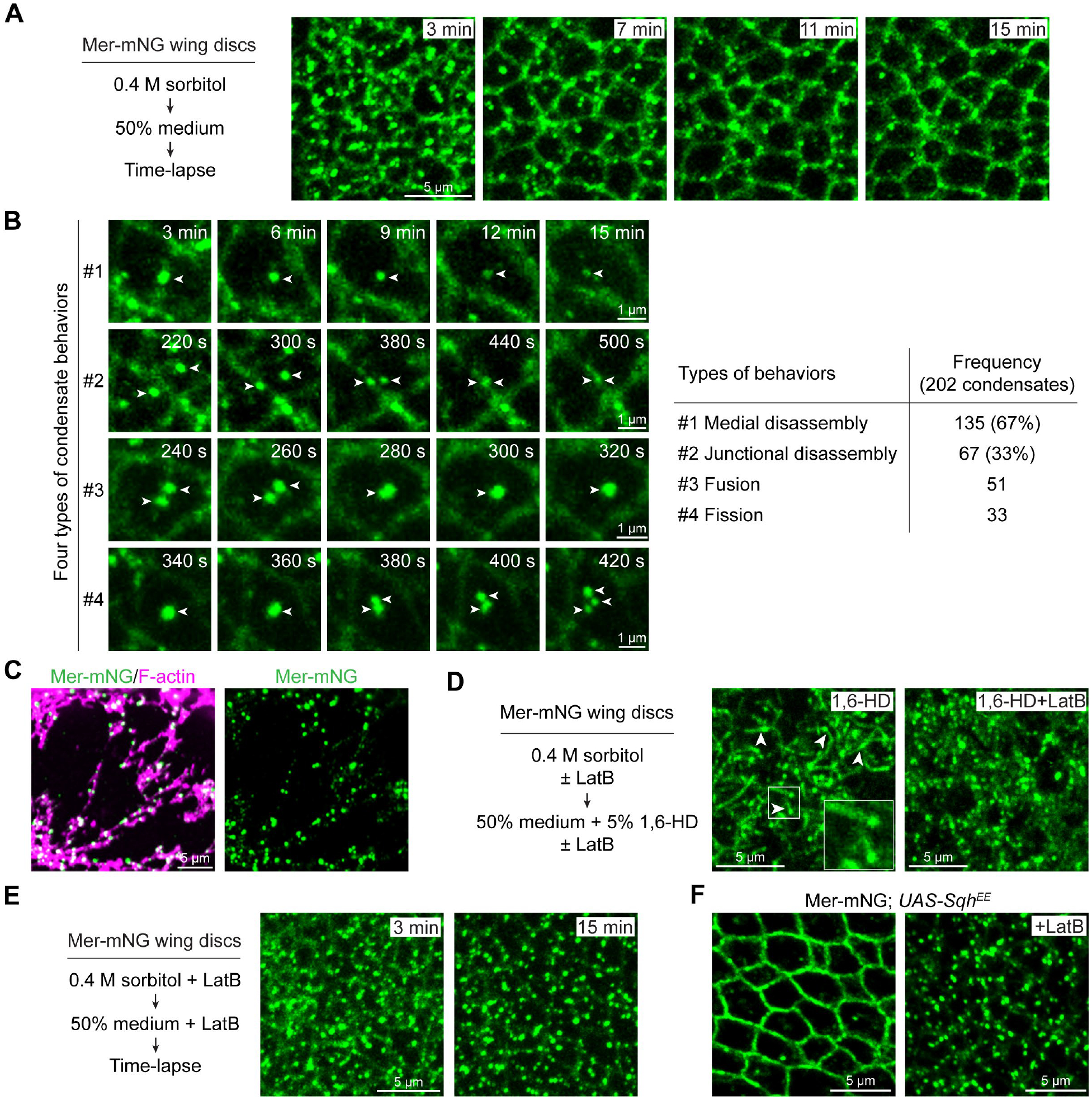
Cytoskeletal tension promotes a physical state transition and disassembly of Mer condensates. (**A**) Time-lapse images showing the disassembly of Mer condensates and relocalization of Mer from the medial apical cortex to the cell junction upon acute elevation of cytoskeletal tension. Wing discs expressing Mer-mNG were incubated in Schneider’s *Drosophila* medium (SDM) with 0.4 M sorbitol for 30 minutes and transferred to 50% SDM (SDM diluted 1:1 with water) before live-cell time-lapse imaging. (**B**) Representative time-lapse images showing distinct Mer condensate behaviors in (A). Quantification of the number of each behavior is shown to the right, *n* = 202 condensates from 105 cells. (**C**) Adult ovaries expressing Mer-mNG were lysed in RIPA buffer followed by fixation and phalloidin staining. Note that the detergent-resistant Mer condensates were all localized on phalloidin-positive F-actin fibers in nurse cells. (**D**) Wing discs expressing Mer-mNG were incubated in SDM with 0.4 M sorbitol for 30 minutes and transferred to 50% medium with 1,6-hexanediol (1,6-HD) for 20 minutes before imaging. Experiments with and without 2 μM LatB were conducted. Note the elongated fibers extending from Mer condensates (representative marked by arrowheads) under 1,6-hexanediol treatment and the absence of such elongation in the presence of LatB. The boxed area is shown at higher magnification. (**E**) Similar to (A) except that the experiment was conducted in the presence of 2 μM LatB. LatB treatment prevented the loss of Mer condensates induced by hypoosmotic stress (compare to Fig. 6A). More complete time-lapse images are also shown in (fig. S11E). (**F**) Mer-mNG wing discs overexpressing Sqh^EE^ without or with 2 μM LatB treatment for 15 minutes before imaging. Note that LatB treatment prevented the loss of medial apical Mer condensates induced by Sqh^EE^ overexpression.

Live-cell time-lapse imaging revealed that Mer condensates in hyperosmotic medium were highly static (fig. S11D). Shifting wing imaginal discs from a hyperosmotic to a hypoosmotic environment caused a rapid transition of Mer from a non-dynamic to a highly dynamic state (Fig. 6, A and B). This transition of physical state eventually resulted in the disassembly of medial apical Mer condensates and relocalization of Mer from the medial apical cortex to the cell junction (Fig. 6A). By time-lapse tracking of 202 Mer condensates from 105 wing disc cells, we identified distinct behaviors of individual Mer condensates during this transition. 135 out of 202 condensates disassembled at the medial apical cortex and 67 out of 202 condensates moved laterally on the cell cortex from medial apical to junctional regions before disassembling at the cell junction (Fig. 6B). We also observed frequent fission and fusion of Mer condensates at the medial apical cortex, indicative of acquisition of liquid-like properties, during this transition (Fig. 6B).

Because NF2 binds F-actin through its N-terminal FERM domain (51–54), we wondered whether physical association between F-actin and Mer condensates might have a role in tension-induced Mer relocalization and condensate disassembly, for example, by directly applying a pulling force on the condensates. Indeed, all Mer condensates were localized on F-actin filaments (Fig. 6C). To reveal whether these condensates were under a pulling force, we treated wing imaginal discs under hypoosmotic stress (to elevate cytoskeletal tension) with 1,6-hexanediol, which interferes with weak hydrophobic interactions (22), as a way to soften Mer condensates and then examined the morphology of the condensates under increased cell tension. In agreement with the notion that Mer condensates are pulled by F-actin cytoskeleton, Mer condensates elongated after 1,6-hexanediol treatment under hypoosmotic stress, reminiscent of melting glass being stretched (Fig. 6D). This elongated morphology was not observed when F-actin cytoskeleton was disrupted by Latrunculin B (LatB), suggesting a role for F-actin in pulling the condensates (Fig. 6D). Further supporting F-actin’s involvement in tension-induced condensate disassembly, LatB treatment prevented the loss of medial apical Mer condensates induced by hypoosmotic stress (Fig. 6E and fig. S11E) or Sqh^EE^ overexpression (Fig. 6F).

## The solid-like material properties protect Mer condensates against external perturbations

**Fig. 7.**
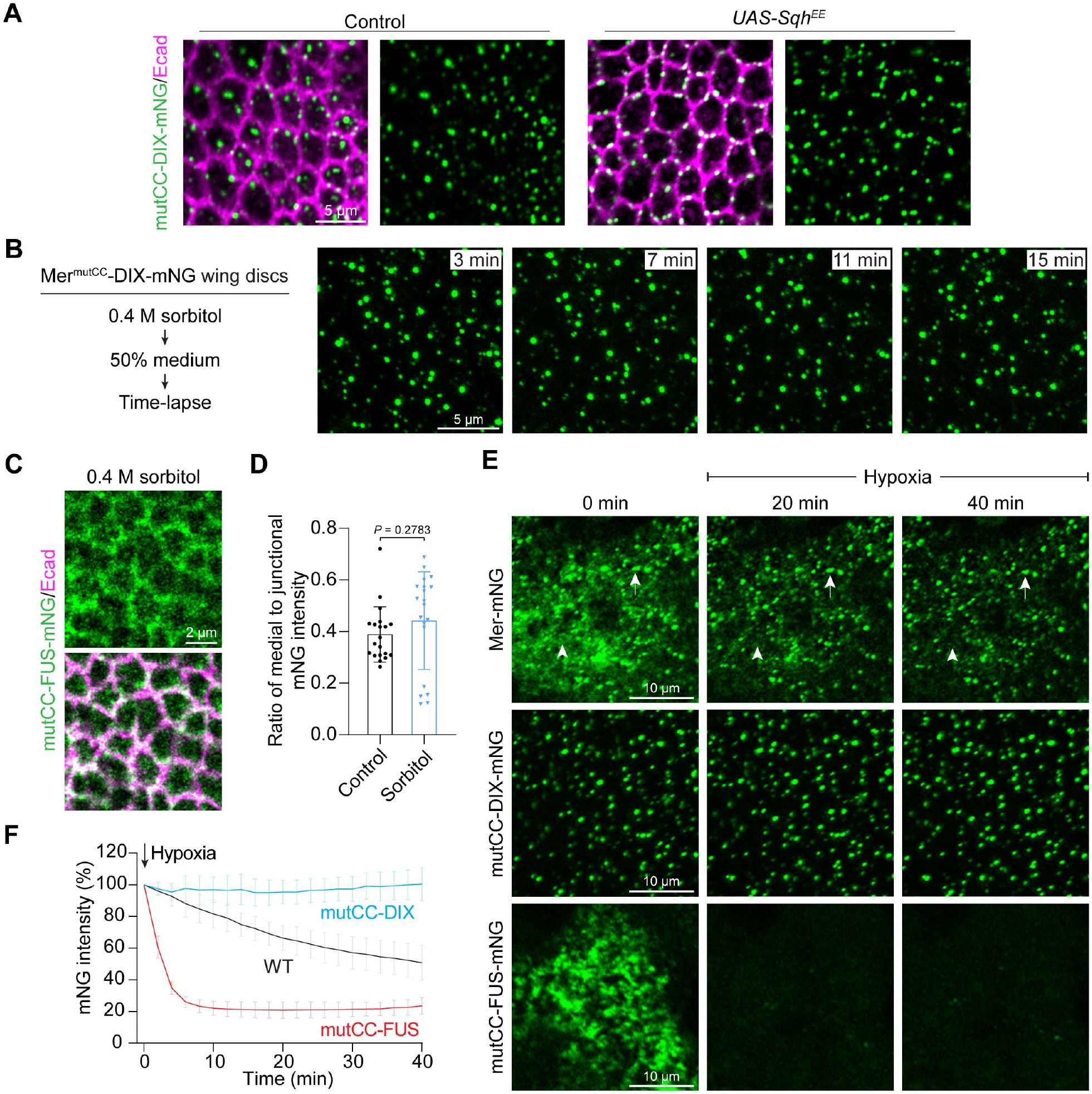
Solid-like material properties protect Mer condensates against external perturbations. (**A**) Wing discs of Mer^mutCC^-DIX-mNG with or without Sqh^EE^ overexpression were stained for Ecad. Note that Mer^mutCC^-DIX condensates were relocalized to the cell junction but were not disassembled in Sqh^EE^-expressing cells. (**B**) Time-lapse images showing that hypoosmotic stress failed to disassemble Mer^mutCC^-DIX condensates (compare to wild-type Mer in Fig. 6A). Wing discs expressing Mer^mutCC^-DIX-mNG were incubated in SDM with 0.4 M sorbitol for 30 minutes and transferred to 50% SDM before live-cell imaging. (**C**) Wing discs expressing Mer^mutCC^-FUS-mNG were incubated in SDM with 0.4 M sorbitol for 30 minutes and stained for Ecad. (**D**) The ratio of medial apical mNG intensity to junctional mNG intensity in Mer^mutCC^-FUS-mNG wing discs incubated in SDM or SDM with 0.4 M sorbitol is quantified. Data are represented as mean ± s.d., *n* = 20, unpaired two-tailed Student’s *t*-test. (**E**) Time-lapse images of ovarian nurse cells expressing wild-type Mer, Mer^mutCC^-DIX-mNG or Mer^mutCC^-FUS-mNG in stage 10 egg chamber under hypoxia. (**F**) Quantification of total mNG fluorescence intensity per unit area in (E). Data are represented as mean ± s.d., *n* = 10 (WT), *n* = 14 (DIX), *n* = 12 (FUS).

To further corroborate our model that cytoskeletal tension promotes a physical state transition and disassembly of Mer condensates, we examined the behavior of the constitutively solid-like Mer^mutCC^-DIX and the constitutively liquid-like Mer^mutCC^-FUS in response to cytoskeletal tension. In contrast to wild-type Mer, increased cytoskeletal tension resulting from either Sqh^EE^-overexpression or hypoosmotic stress failed to disassemble the Mer^mutCC^-DIX condensates (Fig. 7, A and B; compare to Fig. 5D and Fig. 6A). Conversely, whereas hyperosmotic stress (decreased cytoskeletal tension) promoted the formation of medial apical condensates and a loss of junctional wild-type Mer, it had no effect on the liquid-like Mer^mutCC^-FUS, which remained predominantly localized to cell junctions (Fig. 7, C and D). Thus, the solid-like Mer^mutCC^-DIX is more resistant and the liquid-like Mer^mutCC^-FUS is more sensitive to tension-mediated condensate disassembly. We infer from these findings that the material properties of Mer condensates might be set to allow facile regulation by changes of cytoskeletal tension.

The results above showing that the constitutively solid-like Mer^mutCC^-DIX condensates were protected from cytoskeletal tension-induced condensate disassembly led us to test whether the solid-like material properties of Mer condensates might confer a broader resistance to other stress conditions. Given that the formation of Mer condensates is critically regulated by plasma membrane concentration of PI4P, we examined how acute changes of PI4P abundance might impact the behavior of the various Mer mutants with different material properties. We induced hypoxia, which causes acute depletion of plasma membrane PI4P that can be fully recovered upon reoxygenation (55). On nurse cell membranes from stage 10 egg chambers under normoxia condition, wild-type Mer was detected as solid-like condensates as well as non-condensed signals, the solid-like Mer^mutCC^-DIX formed only condensates, and the liquid-like Mer^mutCC^-FUS formed condensates and non-condensed signals (Fig. 7E). After induction of hypoxia, all solid-like condensates, whether derived from wild-type Mer or Mer^mutCC^-DIX, remained stably associated with the cell cortex (Fig. 7, E and F). In contrast, the non-condensed signals from wild-type Mer and all Mer^mutCC^-FUS signals (both condensed and non-condensed) rapidly dissociated from the plasma membrane upon hypoxia (Fig. 7, E and F). Thus, although PI4P is required for Mer plasma membrane association and condensate formation, only the preformed solid-like Mer condensates on the plasma membrane were resistant to acute depletion of PI4P under hypoxia. Thus, the solid-like material properties may protect Mer condensates against external perturbations such as high tension and hypoxia.

## Discussion

The responses of the Hippo pathway to diverse cues are central to its function as a regulator of tissue growth and homeostasis, yet the underlying molecular mechanisms remain poorly understood (11–15). LLPS of Hippo pathway upstream regulators KIBRA, AMOT, and SLMAP provides a simple mechanism for pathway regulation by certain physiological signals such as osmotic stress and cell confluency (16, 24). In contrast to these liquid-like condensates, we report here that Merlin functions as solid-like condensates in regulating Hippo signaling. We further identify plasma membrane PI4P as a key regulator of Merlin condensation, which in turn can be antagonistically regulated by the Hippo-activator Pez and the Hippo-inhibitor cytoskeletal tension. These findings uncover an essential role for solid-like condensates in normal physiology and provide a paradigm for their regulation *in vivo*.

Despite many previous reports of solid-like biomolecular condensates, the functional importance of their solid-like material properties has rarely been probed, especially in physiological contexts (5–7). By engineering Mer mutants that lock the condensates into a solid- or liquid-like state and assaying their *in vivo* activity, we provide evidence that the solid-like material properties of Mer condensates are essential for their medial apical localization and physiological function. This strategy may be generally adapted to relate the material properties of other biomolecular condensates to their biological function. Our analyses further reveal that the solid-like material properties can protect the Mer condensates against external perturbations such as high tension and hypoxia, indicating that solid-like biomolecular condensates may provide a general mechanism to maintain signaling stability against environmental fluctuations. Given the antagonistic regulation of Mer condensation by Pez and cytoskeletal tension, solid-like condensates can apparently be bidirectionally regulated to fulfill different physiological needs. Indeed, by monitoring protein dynamics upon acute increases of cytoskeletal tension, we could directly observe a physical state transition of Mer condensates from a non-dynamic solid-like state to a dynamic liquid-like state as the condensates moved laterally to the cell junction, eventually leading to disassembly of medial apical Mer condensates and an increase of junctional Mer. Our results showing that this transition requires intact F-actin provide a mechanistic explanation for the well-documented effect of F-actin inhibitors in promoting Hippo signaling (20, 56–58).

Our demonstration that the medial apical pool and the junctional pool of PI4P act antagonistically to regulate Hippo signaling in polarized epithelia provides a potential explanation for the conflicting roles of PI4KIIIα, the major plasma membrane PI4P-synthesizing enzyme, in the literature. On the one hand, *PI4KIIIα* was identified as a positive regulator of Hippo signaling from unbiased chemical mutagenesis screen in *Drosophila* (59), which is consistent with our current study showing a requirement for *PI4KIIIα* in PI4P-mediated Mer condensation. On the other hand, studies in cultured mammalian cells implicated the mammalian PI4KIIIα homologue (PI4KA) as a negative regulator of Hippo signaling (60). We suggest that such discrepancy could result from the different cellular architecture in nonpolarized cultured cells versus normal tissues, as only the latter exhibit features of polarized epithelia such as apical-basal polarity and adherens junction. Because mutations in human PI4KA and NF2 cause overlapping neurodevelopmental defects such as hypomyelination and cataracts (21, 61–66), PI4KA-related disorders might be driven by defective Hippo signaling as occurs in Neurofibromatosis type II.

## Materials and Methods

### *Drosophila* stocks and genetics

*Drosophila melanogaster* (RRID:NCBITaxon_7227) crosses and staging were done at 25 °C. The following strains have been described previously: *UAS*-*kibra*-*GFP* (34), *sktl*^*Δ5*^ (67), *PI4KIIIα*^*FQ88*^ (59), *ex*^*e1*^ (68), *mer*^*4*^ (69), *Hpo*-*YFP* (23), *UAS*-*Sqh*^*E20E21*^ (70), *cpa*^*69E*^ (71), *Wts*-*GFP* (*Mi{MIC}wts*^*MI05605*^) (72). RNAi lines were obtained from Vienna *Drosophila* Resource Center: *UAS*-*ex RNAi* (VDRC 22994), *UAS*-*mer RNAi* (VDRC 7161), *UAS*-*PI4KIIIα RNAi* (VDRC 105614). The following strains were generated in this study: *tub*-*mer*-*mNeonGreen* (attP40), *tub*-*mer*^*mutCC*^-*mNeonGreen* (attP40), *tub*-*mer*^*mutCC*^-*DIX*-*mNeonGreen* (attP40), *tub*-*mer*^*mutCC*^-*FUS*-*mNeonGreen* (attP40), *tub*-*GFP*-*2xOsh2*-*PH* (attP2), *tub*-*GFP*-*PLCδ1*-*PH* (attP2), *tub*-*mer*-*HA* (attP40), *tub*-*mer*-*HA* (attP2), *tub*-*mer*^*3A*^-*HA* (attP2), *tub*-*ex* (attP2), *UAS*-*Pez*-*HA* (attP2), *Pez*^*3*^. All transgenic flies were created by phiC31-mediated site-specific transformation. The genotypes for all experimental crosses are listed in Supplementary Table 1.

Lethality rescue experiments were performed by crossing male flies of *tub*-*mer*-*mNeonGreen* (or *tub*-*mer*-*HA*) to *mer*^*4*^*/FM7c* females to give *mer*^*4*^*/y; tub*-*mer*-*mNeonGreen/+* and *FM7c/y; tub*-*mer*-*mNeonGreen/+* male progenies and *mer*^*4*^*/+; tub*-*mer*-*mNeonGreen/+* and *FM7c/+; tub*-*mer*-*mNeonGreen/+* female progenies. The expected number of an 100% rescue would be the total number of female progenies divided by two. The number of observed *mer*^*4*^*/y; tub*-*mer*-*mNeonGreen/+* males was then divided by this number to yield a 69% of rescue.

Mosaic wing discs or ovaries were generated using the FLP/FRT system. For clonal induction in wing discs, heat shocks were carried out 2–4 days after egg-laying in a 37 °C water bath for 1 hour before returning the larvae to 25 °C. Wandering third instar larvae were dissected for immunostaining. For clonal induction in adult ovaries, heat shocks were carried out 2 days after eclosion in a 37 °C incubator for 2 h before returning the flies to 25 °C. Ovaries were dissected 2 days later for immunofluorescence experiments.

The eyeless-FLP/recessive cell lethal technique was used to screen for enhancer mutants of the *ex*^*1*^ overgrowth phenotype (73). Briefly, male flies of *w/Y; ex*^*e1*^ *FRT40A* were fed with 25 mM ethyl methanesulfonate (EMS) and crossed to *w; Adv*^*1*^*/CyO* females. Individual *w/Y; ex*^*e1*^ *FRT40A/CyO* males carrying introduced mutations on the FRT chromosome were subsequently crossed to *w eyFLP; l(2)cl*-*L3 Ubi*-*GFP FRT40A/CyO* flies. Mutant flies with enhanced overgrowth phenotype were selected and maintained as stocks for future characterization.

### Adult wing size measurement

The wings of 5–7-day-old adult females were dissected and mounted in Methyl salicylate:Canada Balsam (1:2) on glass slides. Slides were then dried on a 50 °C heat block overnight before imaging on a Leica M205 FA microscope. Wing sizes were measured using ImageJ and statistical analysis was performed using Graphpad Prism software. The wing size of *nub*-*Gal4* flies was used as a reference.

### Immunofluorescence and western blot

*Drosophila* third instar larval wing discs or adult ovaries were dissected and fixed in 4% formaldehyde for 20 min followed by permeabilization in PBS containing 0.3% Triton X-100 (PBST). For ovary staining, intact ovaries were dissociated into individual egg chambers by pipetting through a 200 μL pipette tip before proceeding. Samples were then incubated in blocking buffer (PBST supplemented with 5% goat serum) for 1 hour at room temperature. Primary antibodies were diluted in blocking buffer and incubated with samples overnight at 4 °C. Samples were washed three times in PBST before incubation in secondary antibodies for 1 hour at room temperature. To visualize F-actin, Alexa Fluor 568-conjugated phalloidin was included in the blocking buffer containing secondary antibodies. Samples were then washed three times in PBST before being mounted in VECTASHIELD on glass slides for imaging. The following primary antibodies were used: anti-Mer (74) (1:10000), anti-Sav (1:200) (75), anti-HA (Cell Signaling Technology, #3724, RRID:AB_1549585, 1:500), anti-ECad (*Drosophila* E-Cadherin, Developmental Studies Hybridoma Bank, DCAD2, RRID:AB_528120, 1:100), anti-Cut (Developmental Studies Hybridoma Bank, 2B10, RRID:AB_528186, 1:100).

For immunostaining of NF2 in HEK293T and MDCK cells, cells cultured in chamber slides were fixed in 4% formaldehyde for 15 min, followed by permeabilization in PBST. Fixed cells were then incubated with primary (Cell Signaling Technology Cat# 6995, RRID:AB_10828709, 1:500) and second antibodies similar to the immunostaining of fly tissues.

Western blot was used to evaluate the expression level of endogenous Mer, Mer-mNG, Mer^mutCC^-DIX-mNG and Mer^mutCC^-FUS-mNG. The third instar larval wing imaginal discs were dissected in cold PBS, and 25 wing imaginal discs were immediately lysed by boiling in 20 μL SDS sample loading buffer. Endogenous Mer, Mer-mNG were blotted using the anti-Mer antibody. Mer^mutCC^-DIX-mNG and Mer^mutCC^-FUS-mNG were blotted using the anti-mNG antibody (proteintech, 32F6, RRID:AB_2827566, 1:1000) and anti-Actin antibody (MilliporeSigma, MAB1501, RRID:AB_2223041, 1:1000) in western blot.

### Live-cell imaging of *Drosophila* tissues and S2R+ cells

Live-cell imaging of wing imaginal discs or ovarian nurse cells was carried out as previously described (23). Briefly, wing discs of third instar larvae were dissected in Schneider’s *Drosophila* medium supplemented with 10% FBS and transferred to glass bottom dishes (MatTeK Corporation, P35G-1.5–14-C). For live-cell imaging of nurse cells, ovaries were dissected from well-fed 3–5-day-old adult females. Egg chambers were further separated from the ovarioles and placed on glass bottom dishes with Schneider’s *Drosophila* medium supplemented with 10% FBS and 200 μg/ml insulin. A small fragment of coverslip was then mounted on top of the wing discs or egg chambers with supporting beads (Cospheric, SLGMS-2.5 125–150μm) beneath to prevent tissue from drifting. Imaging was performed on a Zeiss LSM 880 confocal microscope with a 40x/1.3 oil objective. For live-cell imaging of cultured S2R+ cells, cells were seeded on 4-well chambered coverglass and imaged on a Zeiss LSM 880 confocal microscope with a 63x/1.4 oil objective 48 h after transfection.

### 1,6-hexanediol treatment in *Drosophila* tissues

Tissue dissection was performed as described above. To assess the effect of 1,6-hexanediol treatment in nurse cells, adult ovaries were mounted on dishes as described above except that a final concentration of 5% 1,6-hexanediol was included in the culture medium. Images were taken 20 min after mounting the tissue. To assess the effect of 1,6-hexanediol treatment on the morphology of Mer condensates in wing imaginal discs, wing discs were incubated in medium containing 0.4 M sorbitol with or without 2 μM LatB for 15 minutes and transferred to 50% medium containing 5% 1,6-hexanediol (1,6-HD) with or without 2 μM LatB for incubation of 20 minutes before imaging.

### Hypoxia treatment in *Drosophila* ovary

Ovaries from 2-day-old adult females were dissected and imaged according to previously published protocol (76). To ensure sufficient air exchange for samples during the imaging session, dissected ovaries were mounted in halocarbon oil on an air-permeable membrane (YSI Membrane Model #5793, YSI Inc, Yellow Springs, OH) sealed with vacuum grease on a custom-made plastic slide over a 10 × 10 mm^2^ cut-through window. The slide was then mounted in a custom-made airtight micro chamber (~5 cm^3^) for live imaging under a confocal microscope. Oxygen levels inside the chamber were controlled by the flow of a custom O2/N2 gas mixture at a rate of approximately 1–5 cc/s. Images were captured at room temperature (25 °C) on a Nikon A1 confocal microscope (Plan Fluo 60x oil objective, NA = 1.3) using NIS-Elements AR software.

### Measurement of apical, medial and junctional fluorescence intensity in wing disc cells

Measurement of fluorescence intensity in wing disc cells was performed as previously described (77). All measurements were performed in ImageJ. To measure apical fluorescence intensity, area including the medial apical cortex and the cell junction in a single cell was selected on maximum projections using the freehand selection tool after a single background subtraction (rolling ball radius, 200 pixels). The mean fluorescence intensity of the selected area was then measured. Measurement of junctional fluorescence intensity was performed on maximum projections of immunofluorescence images using the freehand line tool (with a width line of six pixels) after a single background subtraction (rolling ball radius, 200 pixels). To measure the medial fluorescence intensity, apical area excluding the cell junction was selected on maximum projections using the freehand selection tool after a single background subtraction (rolling ball radius, 200 pixels). The mean fluorescence intensity of the selected line and area was then measured. For comparing intensity values in cells of different genetic background, fluorescence intensity in cells of the indicated genetic background was normalized to the mean intensity in wild-type cells in the same disc. Quantification is for a cell’s apical fluorescence intensity unless otherwise indicated.

### Measurement of diameter and aspect ratio

The diameter and aspect ratio of Mer-mNG condensates in S2R+ cells were measured using ImageJ. S2R+ cells transfected with tub-Mer-mNG were imaged 48 h after transfection on a Zeiss LSM 880 confocal microscope with a 63x/1.4 oil objective. The length of the major and minor axis of condensates was measured, and the aspect ratio was calculated as the ratio of the major axis to the minor axis. The length of the major axis was used to represent the diameter of a condensate.

### Prediction of coiled-coil domain

Prediction of coiled-coil heptad repeats was performed using Marcoil (78), https://toolkit.tuebingen.mpg.de/tools/marcoil.

### Constructs and cloning

For ubiquitous expression of fusion proteins in fly, the UAS cassette of pUASTattB was replaced by a tubulin promoter to generate the vector pTUBattB as described before. To express mNeonGreen (mNG, licensed from Allele Biotechnology) fusion proteins, a *Drosophila* codon-optimized mNG (synthesized by Genscript) was inserted into pTUBattB to generate pTUBattB-mNG. Wild-type Mer or Mer^mutCC^ (synthesized by Genscript) was then inserted before mNG to generate tub-Mer-mNG or tub-Mer^mutCC^-mNG. The DIX (human Dishevelled amino acids 12–93, amplified from Addgene plasmid 24802 (79)) and FUS LC (human Fused in sarcoma amino acids 1–214, amplified from Addgene plasmid 44985 (80)) sequence were inserted between Mer^mutCC^ and mNG to generate tub-Mer^mutCC^-DIX-mNG and tub-Mer^mutCC^-FUS-mNG. To express PI4P and PIP2 sensors, GFP was first inserted into pTUBattB followed by insertion of 2xOsh2-PH (81) (amplified from Addgene plasmid 36095) or PLCδ1-PH (82) (amplified from Addgene plasmid 21179) after GFP to generate tub-GFP-2xOsh2-PH and tub-GFP-PLCδ1-PH. To ubiquitously express other proteins, cDNA with or without HA tag was directly inserted into pTUBattB. Mer K67A, K70A and K288A triple mutation was generated by site-directed mutagenesis. UAS-Pez-HA was generated by inserting Pez with HA tag at its C-terminus into pUASTattB.

For protein expression in S2R+ cells, the pTUBattB constructs described above were used to express mNG fusion proteins and phosphatidylinositol phosphate sensors. mNeonGreen in the tub-Mer-mNG construct was replaced by mScarlet-I to express Mer-mScarlet. For expression of PTPN14-mEGFP in MDCK cells, PTPN14-mEGFP was first cloned into pENTR4 using NEBuilder HiFi DNA Assembly and subsequently transferred to pCW57.1 using Gateway LR Clonase.

The pET28a vector or pRSFDuet vector was used to express recombinant proteins in *E.coli* cells. Mer-CFP and Mer^mutCC^-CFP (The monomeric variant of CFP, mTurquoise2, was used to prevent dimer formation), NF2-mEGFP and mScarlet-Hpo were cloned into the NdeI/XhoI sites on pET28a using NEBuilder HiFi DNA Assembly. For co-expression in *E.coli* cells, Sav and mScarlet-Hpo were sequentially cloned into pRSFDuet at BamHI/NotI sites and NdeI/XhoI sites.

### Cell Culture, plasmid transfection, lentivirus packaging and infection

*Drosophila* S2R+ cells (Drosophila Genomic Resource Center, Cat#150, RRID:CVCL_Z831) were cultured in Schneider’s *Drosophila* medium supplemented with 10% fetal bovine serum (FBS) and 1% Antibiotic-Antimycotic at 25 °C. MDCK (ATCC Cat# CCL-34, RRID:CVCL_0422) and HEK293T (ATCC Cat# CCL- 3216, RRID:CVCL_0063) cells were cultured in DMEM medium supplemented with 10% fetal bovine serum and 1% penicillin/streptomycin at 37 °C in a humidified atmosphere with 5% CO_2_. Plasmid transfection was conducted using Effectene transfection reagent according to the manual (Qiagen, Cat# 301425).

To express PTPN14-mEGFP in MDCK cells, an inducible stable cell line was generated by lentivirus infection. HEK293T cells seeded in a 6-well plate at 90% confluency were co-transfected with pCW57.1-PTPN14-mEGFP, pMD2.G and psPAX2 using Effectene. Lentivirus particles were harvested by filtering the culture medium through 0.45 μm sterilized Millex-HV Syringe Filters (Millipore) 48 h after transfection. Lentivirus particles were added to the MDCK cells with 2 μg/mL polybrene. Lentivirus-containing medium was replaced by fresh medium 48 h after infection. Stable expression cells were selected with 2 μg/mL puromycin. To induce PTPN14-mEGFP expression, stable expression MDCK cells were 1:1 mixed with wild-type cells followed by adding 500 ng/mL doxycycline. Cells were then fixed 24 h later for immunostaining.

### Chemical crosslinking

S2R+ cells were resuspended in PBS (pH 8.0) 48 h after transfection and washed three times with ice-cold PBS (pH 8.0). Cells were then incubated with PBS (pH8.0) containing 0.05 mM (full-length Mer) or 0.25 mM (Mer fragments) disuccinimidyl suberate (DSS, Sigma A39267) at room temperature for 30 min with gentle rocking. Tris-HCl (pH7.5) was added to the cell suspension to a final concentration of 20 mM and incubated for 15 min at room temperature to quench the crosslinking reaction before the cells were lysed in SDS sample loading buffer for western blot analysis.

### Protein solubility analysis

To assay the solubility of Mer-HA and HA-Kibra expressed in S2R+ cells or HA-NF2 expressed in 293T cells, transiently transfected cells were lysed with TNTE buffer (50 mM Tris-HCl pH 7.4, 150 mM NaCl, 1% Triton X-100, 1 mM EDTA) supplemented with protease inhibitor cocktail for 45 min at 4 °C. Whole cell lysate was then centrifugated at 15,000 rpm for 5 min at 4 °C to separate the soluble (supernatant) and insoluble (pellet) fraction. The pellet was resuspended in an equal volume of TNTE buffer and both fractions were boiled in SDS sample loading buffer for western blot analysis. To assay the solubility of Mer-mNG expressed wing imaginal discs, discs were dissected as described above and lysed with RIPA buffer (50 mM Tris-HCl pH 7.4, 150 mM NaCl, 1% Nonidet P-40, 0.5% sodium deoxycholate, 0.1% SDS) supplemented with protease inhibitor cocktail for 30 min at 4 °C. Tissue lysate was then centrifugated at 15,000 rpm for 5 min at 4 °C to separate the soluble and insoluble fraction. For confocal microscopy analysis, the pellet was resuspended in 10 μL RIPA buffer and loaded onto a glass slide, covered with coverglass before imaging. For western blot analysis, the pellet was resuspended in an equal volume of RIPA buffer and both fractions were boiled in SDS sample loading buffer.

### Expression and purification of recombinant proteins

6xHis-Mer-CFP, 6xHis-Mer^mutCC^-CFP, 6xHis-NF2-mEGFP, 6xHis-mScarlet-Hpo proteins and 6xHis-Sav/mScarlet-Hpo protein complex were produced in *Escherichia coli* (*E.coli*) BL21(DE3) cells. Bacteria cells were cultured in LB medium to log phase and protein expression was induced by 200 μM IPTG (OD_600_=0.8) overnight at 16 °C. Cells were collected and resuspended in lysis buffer (50 mM Tris-HCl pH 7.5, 500 mM NaCl, 5 mM imidazole, 1 mM DTT) supplemented with protease inhibitors and lysed by sonication. The cell lysate was centrifuged at 20,000 rpm for 45 min at 4 °C. The supernatant was incubated with Ni-NTA agarose for 2 h and agarose beads were washed with wash buffer (50 mM Tris-HCl pH 7.5, 500 mM NaCl, 50 mM imidazole and 1 mM DTT). Proteins were eluted with elution buffer (50 mM Tris-HCl pH 7.5, 500 mM NaCl, 150 mM imidazole and 1 mM DTT) and then desalted with Disposable PD 10 Desalting Columns (GE Healthcare) against stocking buffer (50 mM Tris-HCl pH 7.5, 500 mM NaCl and 1 mM DTT). Purified proteins were snap frozen in aliquots and stored at −80 °C. Protein concentrations were measured by Bradford protein assay.

### *In vitro* phase separation assay

Frozen aliquots of purified proteins were thawed at room temperature and centrifuged at 15,000 rpm for 5 min to remove aggregates. Phase separation was induced by adjusting NaCl concentration to 150 mM and protein concentration to 0.3 μM (Mer-CFP and Mer^mutCC^-CFP) or 0.5 μM (NF2-mEGFP). Phase separation reaction was assembled in Eppendorf tube. 30 μL of the reaction was loaded onto a MatTek glass bottom dish and incubated in a humidified chamber before imaging on a Zeiss LSM 880 confocal microscope with a 63x/1.4 oil or 40x/1.3 oil objective. To assess the incorporation of mScarlet-Hpo or Sav/mScarlet-Hpo complex into Mer-CFP condensates, 0.3 μM of the purified proteins were added to the reaction 2 h after the induction of Mer-CFP condensation. Condensates were then imaged 30 min later. To evaluate the effect of 1,6-hexanediol on *in vitro* reconstituted condensates, 3 mL of salt buffer (50 mM Tris-HCl pH 7.5, 150 mM NaCl) containing 0% (control), 5% (for Mer-CFP) or 10% (for NF2-mEGFP) 1,6-hexanediol was gently added to the phase separation reaction on the MatTek dishes. Condensates were then imaged after incubation for 15 min. Quantification of the number of Mer-CFP condensates was performed using ImageJ in a 27 μm × 27 μm area.

### Protein-lipid overlay assay

Protein-lipid overlay assay was performed using membrane lipid strips (Echelon Biosciences, P-6002) according to the protocol from the manufacturer. Briefly, lipid strips were incubated with blocking buffer (TBS with 0.1% Tween-20 and 1% non-fat milk) overnight at 4 °C. Purified proteins were then added to the blocking buffer to a final concentration of 1 μg/mL and incubated with the lipid strips at room temperature for 1 h. Lipid strips were washed three times with TBST (TBS with 0.1% Tween-20) and subsequently incubated with HRP-conjugated anti-6xHis antibody (Thermo Fisher Scientific, MA1–21315-HRP, RRID:AB_2536989, 1:1000) in blocking buffer at room temperature for 1 h. Strips were then stained similar to regular immunoblotting procedure.

### Cryo-electron Tomography

*in vitro* Mer-CFP condensates were prepared as described above. 3 μL of the phase separation reaction was applied on a glow-discharged 300-mesh lacey carbon grid for the Cryo-ET grid preparation. The grid was then blotted for 4 seconds and immediately plunged froze in liquid ethane by Vitrobot. Tilt-series were collected on an FEI Titan Krios transmission electron microscopy equipped with the Gatan Quantum energy filter and a K3 camera. The stage was tilted range from −60° to −60° using a dose symmetry scheme (83) with an angular increment of 3° per tilt. The images were recorded at 0.5 μm defocus with a Volta phase plate. Alignment of the tilt-series was performed with AreTomo (84) fiducial-less tracking and then reconstructed with simultaneous algebraic reconstruction technique (SART). The tomograms were then subjected to IsoNet (85) for missing-wedge correction and denoising.

### Fluorescence recovery after photobleaching (FRAP) assay

FRAP assays were conducted on a Zeiss LSM 880 confocal microscope at room temperature with a 40x/1.3 oil objective for fly tissues and 63x/1.4 oil objective for S2R+ cells or *in vitro* condensates. For mNG and GFP fusion proteins in fly tissues, a circular region covering the whole condensate was photobleached using the 488 nm laser beam at 100% laser power. Fluorescence images were acquired immediately before and after photobleaching every 5 s for 325 s. For S2R+ cells, fluorescence images were acquired immediately before and after photobleaching every 2 s for 66 s. FRAP images were analyzed using ImageJ. Briefly, the fluorescence intensity of condensates immediately before bleaching and during the recovery period were recorded and normalized against neighboring unbleached condensates with similar intensity. The intensity before photobleaching was set as 100%. Data was plotted using Graphpad prism software. For *in vitro* Mer-CFP condensates, a square region covering half of the condensate was photobleached using the 458 nm laser beam at 100% laser power. To perform FRAP after 100-fold dilution, 3 mL of salt buffer (50 mM Tris-HCl pH 7.5, 150 mM NaCl) was gently added to the phase separation reaction on the MatTek dishes 2 h after induction of phase separation. Images were acquired every minute for 10 minutes.

### Quantification, graphing and statistical analysis

All images were analyzed and quantified using ImageJ. Data was plotted and statistical analysis was performed using Graphpad Prism 9 software.

## Supplementary Material

1

## Data and materials availability:

All data necessary to evaluate the conclusions in this paper are present either in the main text or the Supplementary information files. Additional data supporting the findings of this study are available from the corresponding author upon reasonable request.
