# Supplementary material for: PI4P-mediated solid-like Merlin condensates orchestrate Hippo pathway regulation
Source: Science. Author manuscript; Available in PMC 2025 Mar 31. (PMC11956869; doi:10.1126/science.adf4478)
Supplement: 1 [file NIHMS2068157-supplement-1.pdf]

Science AAAS

Supplementary Materials for

PI4P-mediated solid-like Merlin condensates orchestrate Hippo pathway regulation

Pengfei Guo, Bing Li, Wei Dong, Huabin Zhou, Li Wang, Ting Su, Christopher Carl, Yonggang Zheng, Yang Hong, Hua Deng, Duojia Pan

Correspondence to: hua.deng@utsouthwestern.edu (HD), duojia.pan@utsouthwestern.edu (DP)

This PDF file includes:

Figs. S1 to S11

Table S1

Other Supplementary Materials for this manuscript include the following:

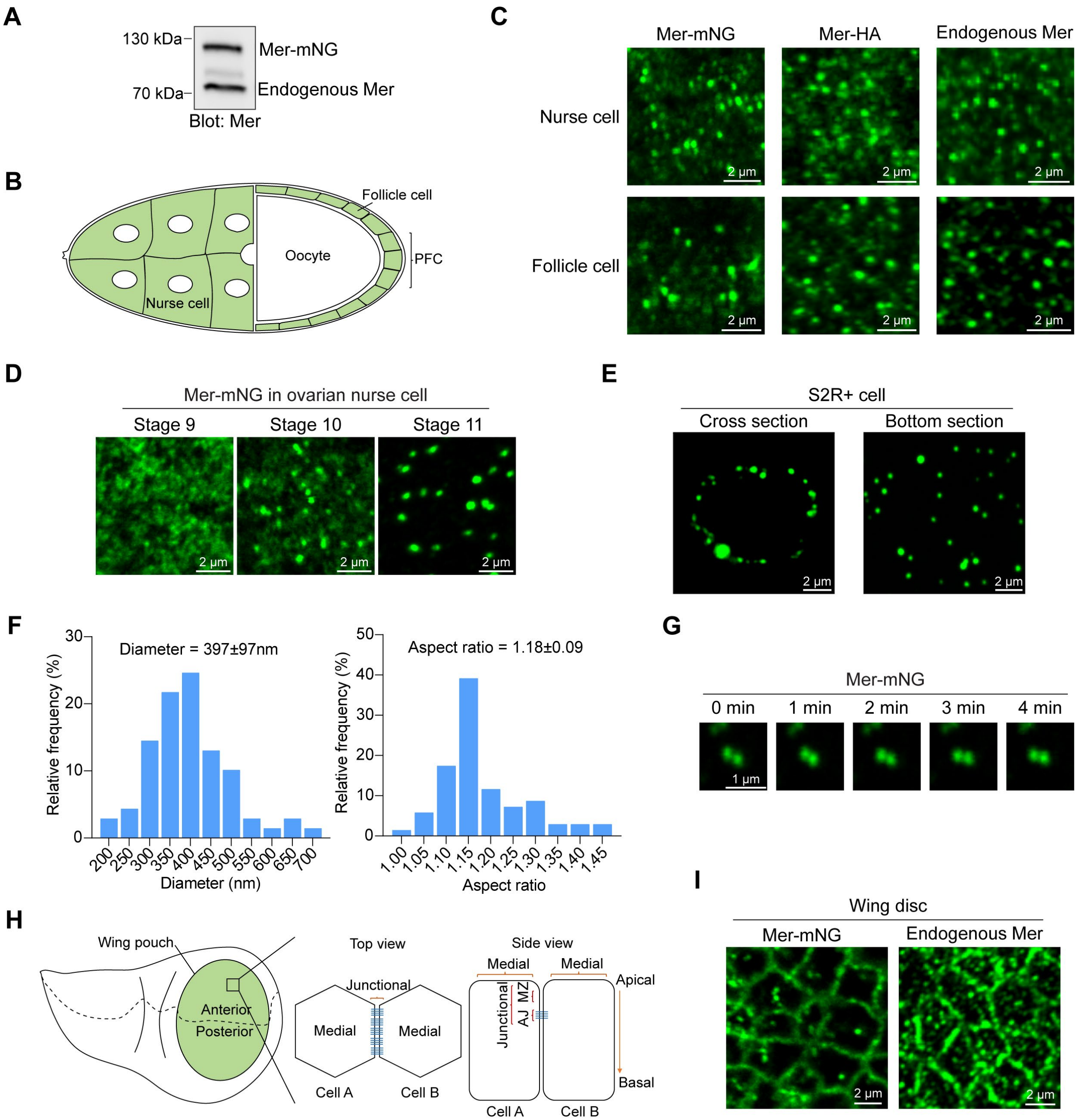

Fig. S1. Mer forms solid-like condensates *in vivo*. (**A**) Wing discs expressing Mer-mNG were analyzed by western blot using anti-Mer antibody. Note that Mer-mNG (upper band) was expressed at comparable level as endogenous Mer (lower band). (**B**) A schematic diagram of a stage 10 egg chamber showing the nurse cells, oocyte and follicle cells. PFC: posterior follicle cell. (**C**) Stage 10 egg chamber nurse cells or follicle cells expressing Mer-mNG, Mer-HA or stained for endogenous Mer. (**D**) Ovarian nurse cells expressing Mer-mNG at the indicated egg chamber developmental stages. (**E)** S2R+ cells expressing Mer-mNG showing the plasma

membrane localization of Mer-mNG condensates. Cross section and bottom surface section are shown. (**F**) Histogram showing the distribution of the aspect ratio and the diameter of Mer-mNG condensates in S2R+ cells, n = 69. (**G**) Time-lapse images showing that two Mer-mNG condensates in a nurse cell did not fuse upon contact. (**H**) A schematic diagram depicting a third instar larval wing imaginal disc. Adult wing develops from the pouch region. The boundary between the anterior and posterior compartments is marked by a dashed line. To the right are sections showing the top view (tangential section) and side view (cross section) of wing disc cells. MZ: marginal zone; AJ: adherens junction. (**I**) Wing discs expressing Mer-mNG or stained for endogenous Mer.

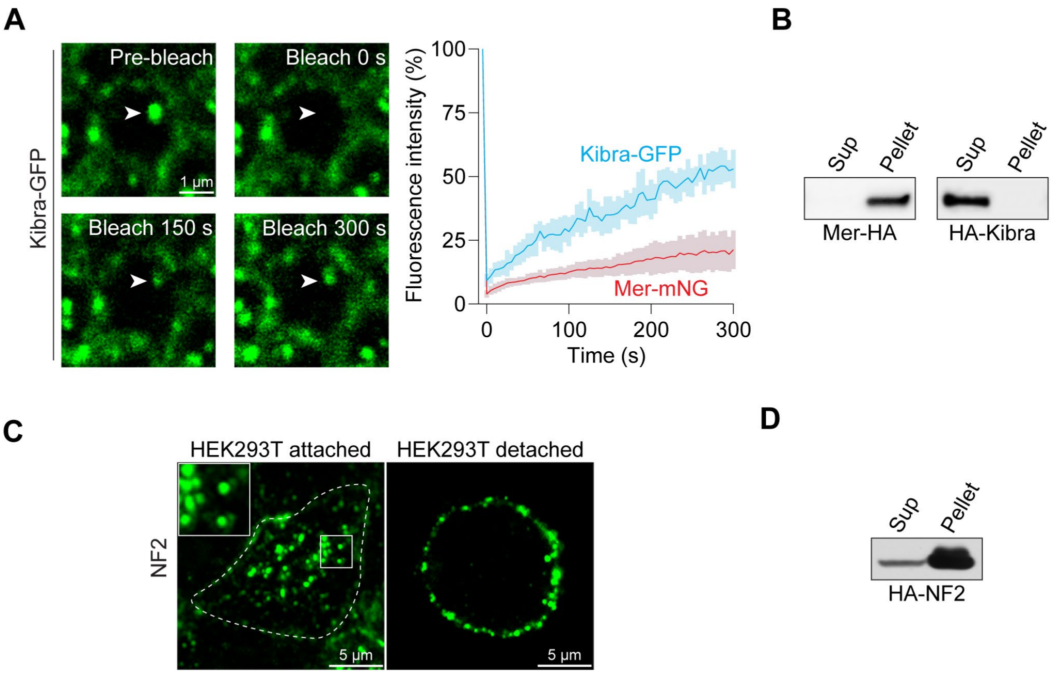

Fig. S2. Mer and NF2 form solid-like condensates *in vivo*. (**A**) FRAP analysis of medial Kibra-GFP condensates in wing discs. Arrowhead marks the photobleached condensate. Quantification of fluorescence intensity is shown to the right. FRAP analysis of medial Mer-mNG (Fig. 1A) is included for comparison. Data are represented as mean ± s.d., $n = 8$. (**B**) Protein solubility analysis showing that Mer-HA but not HA-Kibra expressed in S2R+ cells was insoluble in Triton X-100 lysis buffer. Cell lysates were separated by centrifugation into soluble supernatant (Sup) and insoluble pellet fraction. (**C**) Attached or detached HEK293T cells stained for endogenous NF2 showing the punctate pattern and plasma membrane localization. The focal plane of the plasma membrane was imaged for the attached cell. The boxed area is shown at higher magnification. Cell boundary is outlined by dashed line. (**D**) Protein solubility analysis showing that HA-NF2 expressed in 293T cells was mostly insoluble in Triton X-100 lysis buffer.

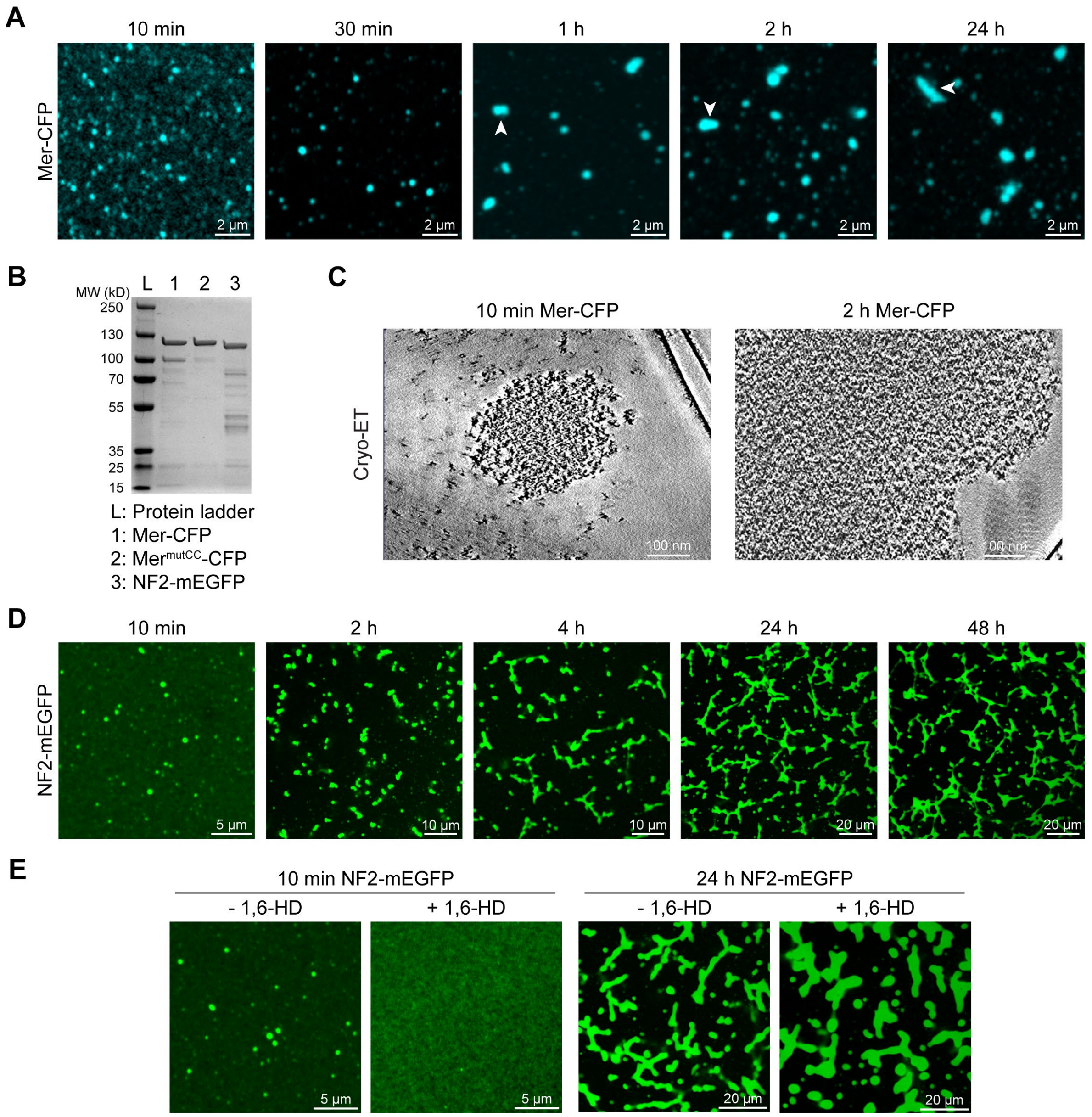

Fig. S3. Mer and NF2 form solid-like condensates *in vitro* that age with time. (**A**) *in vitro* reconstituted Mer-CFP condensates at indicated time points after the induction of phase separation. Arrowheads mark the unresolved fusion intermediates. (**B**) Coomassie blue staining of the indicated purified recombinant proteins. (**C**) Cryo-ET images of *in vitro* reconstituted Mer-CFP condensates at the indicated time points after inducing phase separation. Only a portion of a 2-hr unresolved fusion intermediate is shown due to its large size. (**D**) *in vitro* reconstituted NF2-mEGFP condensates at indicated time points after induction of phase separation. (**E**) *in vitro* reconstituted NF2-mEGFP condensates at the indicated time points after inducing phase separation were treated with or without 10% 1,6-hexanediol for 15 min before imaging.

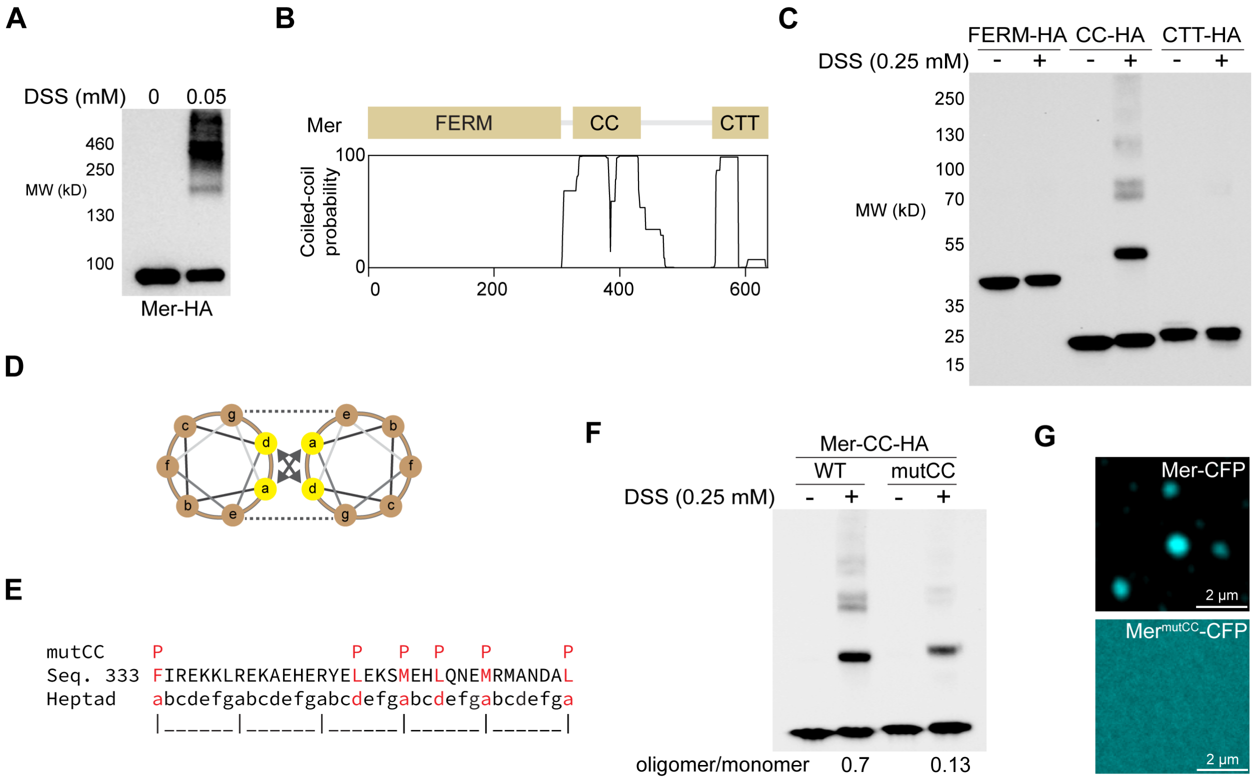

Fig. S4. Mer condensation requires CC domain-mediated oligomerization. (**A**) Crosslinking analysis using the crosslinker disuccinimidyl suberate (DSS) showing the oligomerization of Mer-HA expressed in S2R+ cells. (**B**) A schematic diagram showing the domain organization (top) and the prediction of coiled-coil (CC) domain probability (bottom) of Mer protein. (**C**) Crosslinking analysis using DSS showing oligomerization of the CC domain (aa 310-470) but not the FERM domain (aa 1-330) or the C-terminal tail (CTT) domain (aa 471-635). (**D**) A schematic diagram showing that hydrophobic residues at the a and d positions of heptad repeats mediate the interaction between two coiled-coil α-helices. (**E**) Amino acid sequence of Mer 333-368. The predicted heptad repeats are shown below and the proline mutations in Mer^{mutCC} are shown above. (**F**) Crosslinking analysis showing reduced oligomerization of the mutant (mutCC) coiled-coil domain. The ratio of oligomer to monomer is shown below. (**G**) *in vitro* phase separation assay of Mer-CFP and Mer^{mutCC}-CFP showing that mutation of the coiled-coil domain disrupted Mer condensation.

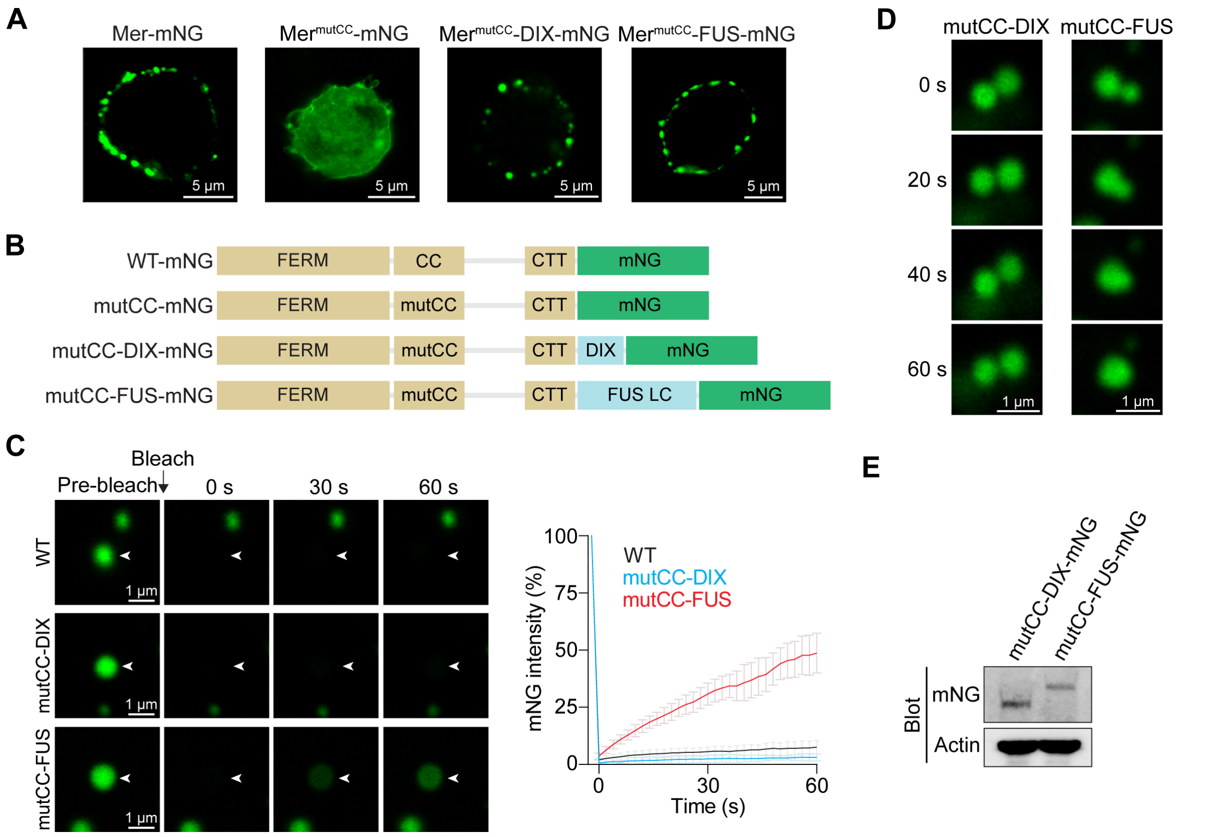

Fig. S5. Characterization of Mer^{mutCC}-DIX and Mer^{mutCC}-FUS condensates. (**A**) S2R+ cells expressing the indicated proteins showing the condensation and plasma membrane association of Mer-mNG; diffusive cytoplasmic localization of Mer^{mutCC}-mNG; restoration of condensation and plasma membrane association of Mer^{mutCC}-FUS-mNG and Mer^{mutCC}-DIX-mNG. (**B**) A schematic diagram showing the domain organization of Mer-mNG, Mer^{mutCC}-mNG, Mer^{mutCC}-FUS-mNG and Mer^{mutCC}-DIX-mNG fusion proteins. (**C**) FRAP analysis of Mer-mNG, Mer^{mutCC}-DIX-mNG and Mer^{mutCC}-FUS-mNG condensates in S2R+ cells. Arrowheads mark the photobleached condensates. Quantification of fluorescence intensity is shown to the right. Data are represented as mean ± s.d., n = 15 for each protein. (**D**) Time-lapse images showing that two Mer^{mutCC}-DIX-mNG condensates did not fuse upon contact whereas two Mer^{mutCC}-FUS-mNG condensates rapidly fused with each other in S2R+ cells. (**E**) Wing discs expressing Mer^{mutCC}-DIX-mNG or Mer^{mutCC}-FUS-mNG were analyzed by western blot using anti-mNG antibody. Note that the two proteins were expressed at comparable level.

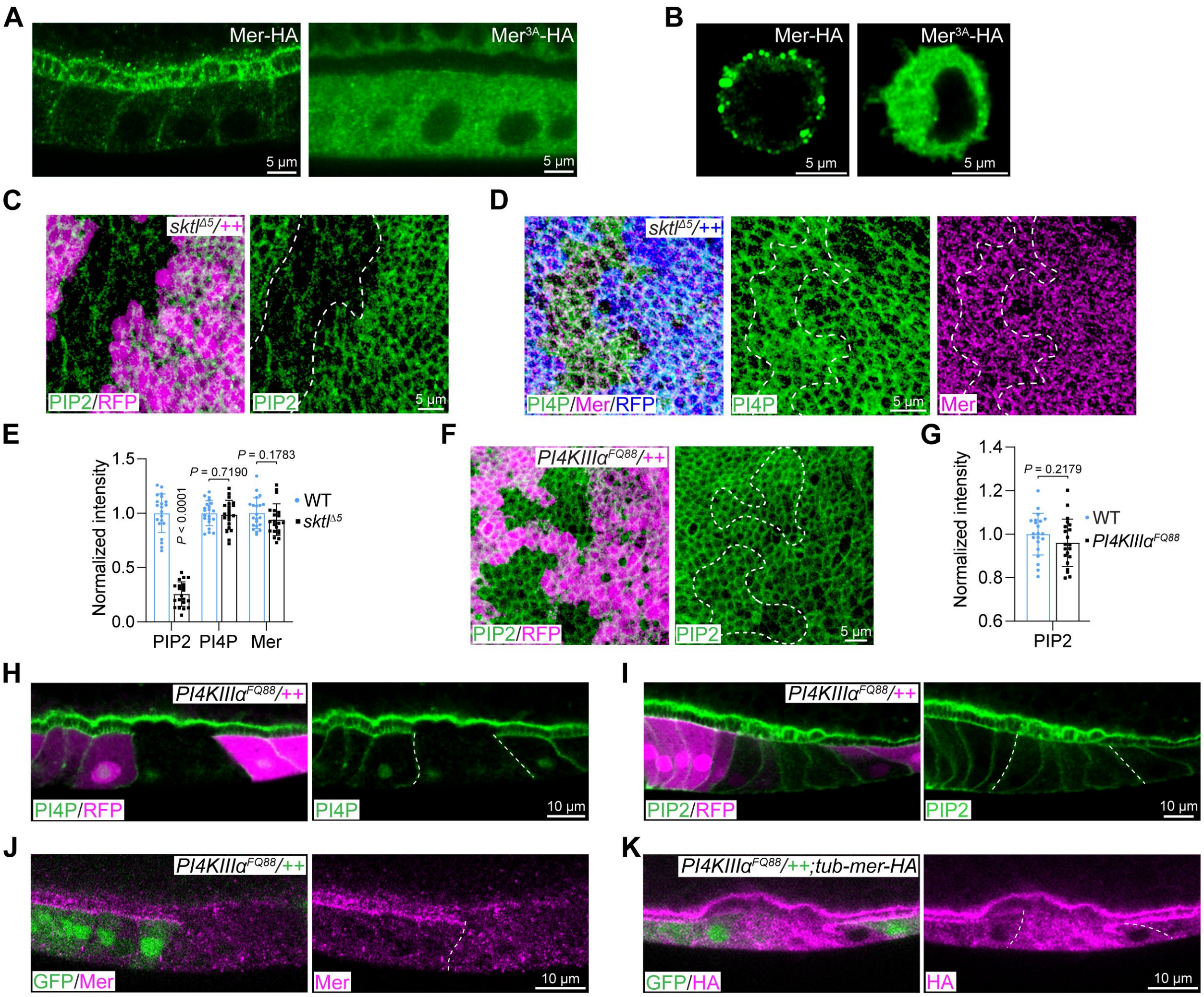

Fig. S6. Plasma membrane association of Mer requires PI4P. (**A** and **B**) Follicle cells (A) or S2R+ cells (B) expressing Mer-HA or Mer^{3A}-HA were stained for HA. Note the mis-localization of Mer^{3A} to the cytoplasm. (**C**) A wing disc containing RFP-negative clones of *sktl*$^{\Delta 5}$ and expressing the PIP2 sensor GFP-PLCδ1-PH. (**D**) A wing disc containing RFP-negative clones of *sktl*$^{\Delta 5}$ and expressing the PI4P sensor GFP-2xOsh2-PH was stained for Mer. (**E**) Quantification of GFP-PLCδ1-PH, GFP-2xOsh2-PH and Mer fluorescence intensity in (C and D). Data are represented as mean ± s.d., $n = 21$, unpaired two-tailed Student's *t*-test. (**F**) A wing disc containing RFP-negative clones of *PI4KIIIα*^{FQ88} and expressing GFP-PLCδ1-PH. (**G**) Quantification of GFP-PLCδ1-PH fluorescence intensity in (F). Data are represented as mean ± s.d., $n = 21$, unpaired two-tailed Student's *t*-test. (**H** and **I**) Follicle cells containing RFP-negative clones of *PI4KIIIα*^{FQ88} and expressing GFP-2xOsh2-PH (H) or GFP-PLCδ1-PH (I). (**J** and **K**)

Follicle cells containing GFP-negative clones of *PI4KIIIα*^{FQ88} were stained for endogenous Mer (J) or an HA epitope from a tub-Mer-HA transgene (K).

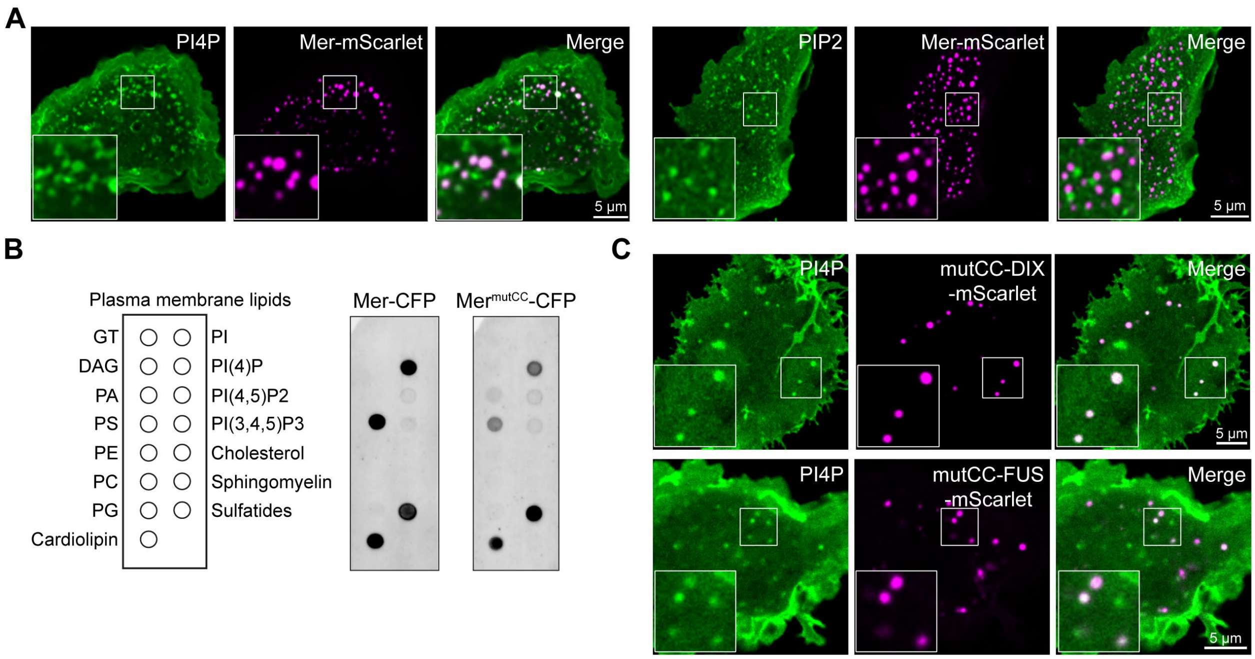

Fig. S7. Oligomerization of Mer promotes PI4P binding. (**A**) S2R+ cells co-expressing Mer-mScarlet with GFP-2xOsh2-PH or GFP-PLCδ1-PH showing the localization of Mer-mScarlet condensates with PI4P-positive but not PIP2-positive microdomains. The boxed area is shown at higher magnification. (**B**) Protein-lipid overlay assay showing the selective binding of purified Mer-CFP to PI4P and the reduced PI4P binding of Mer^{mutCC}-CFP. (**C**) S2R+ cells co-expressing Mer^{mutCC}-DIX-mScarlet or Mer^{mutCC}-FUS-mScarlet with GFP-2xOsh2-PH showing the localization of the two proteins with PI4P-positive microdomains. The boxed area is shown at higher magnification.

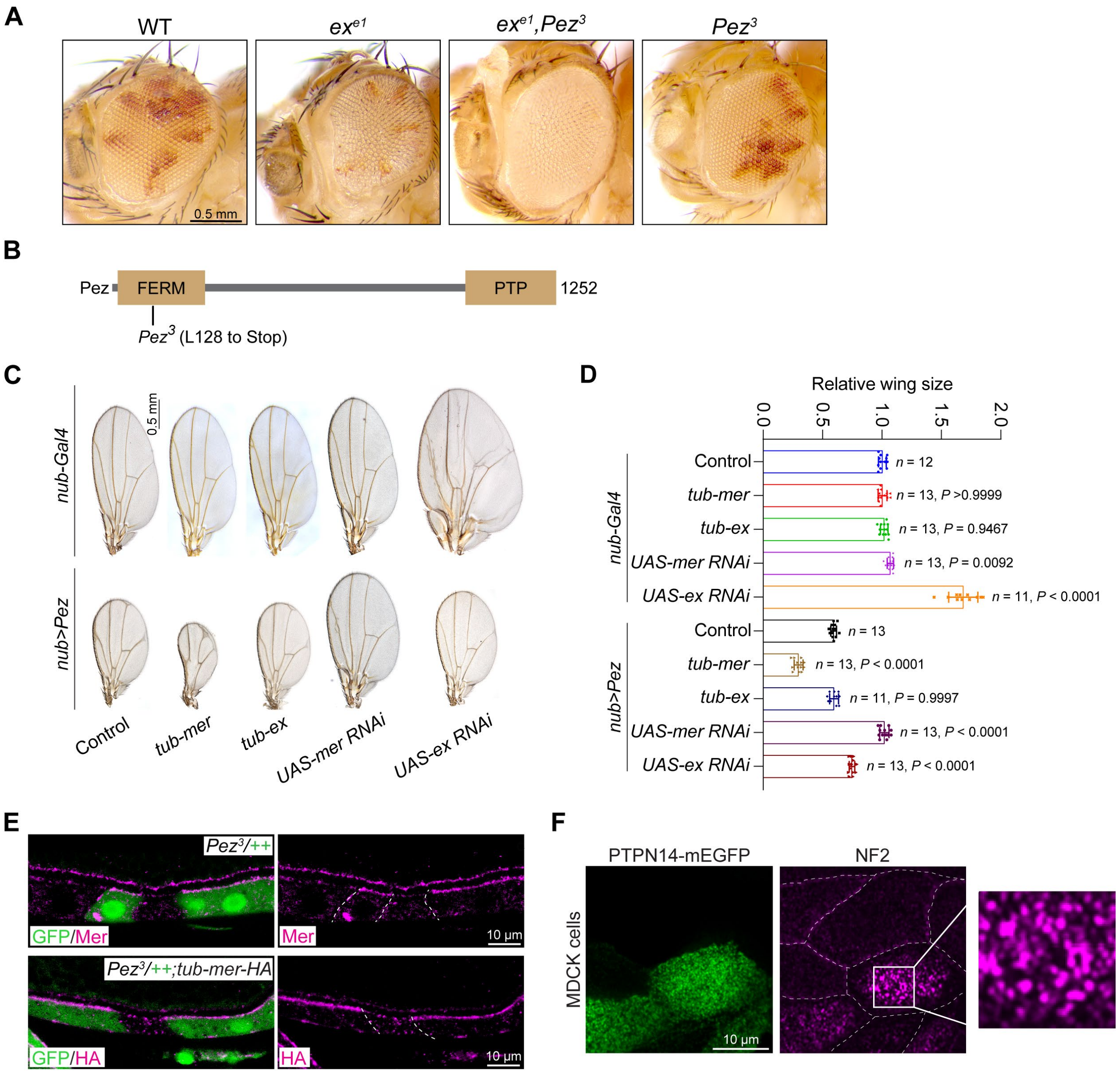

Fig. S8. Pez acts upstream of Mer in the Hippo pathway. (**A**) Adult eyes containing heterozygous (red) and homozygous (white) tissues of the indicated genotypes generated by the ey-FLP/recessive cell lethal technique. Note the increased occupancy of homozygous *ex*^{e1}*, Pez*^{3} double mutant tissues compared to homozygous *ex*^{e1} single mutant tissues in mosaic eyes. (**B**) A schematic diagram showing the domain organization of Pez and the nonsense mutation identified in the *Pez*^{3} mutant. (**C**) Adult wings of *nub-Gal4* or *nub-Gal4;UAS-Pez* flies carrying the indicated transgenes. (**D**) Quantitation of wing size in (C). Data are represented as mean ± s.d., one-way ANOVA with Dunnett's test. (**E**) Follicle cells containing GFP-negative *Pez*^{3} clones were stained for endogenous Mer (top), compared to follicle cells with similarly marked *Pez*^{3}

clones in tub-Mer-HA transgenic flies and stained for HA epitope (bottom). (**F**) MDCK cells stably expressing doxycycline-inducible PTPN14-mEGFP were mixed with wild-type MDCK cells. PTPN14-mEGFP expression was induced by doxycycline and cells were stained for endogenous NF2. Note the medial accumulation of NF2 in PTPN14-overexpressing cells. Cell boundaries are outlined. The boxed area is shown at higher magnification.

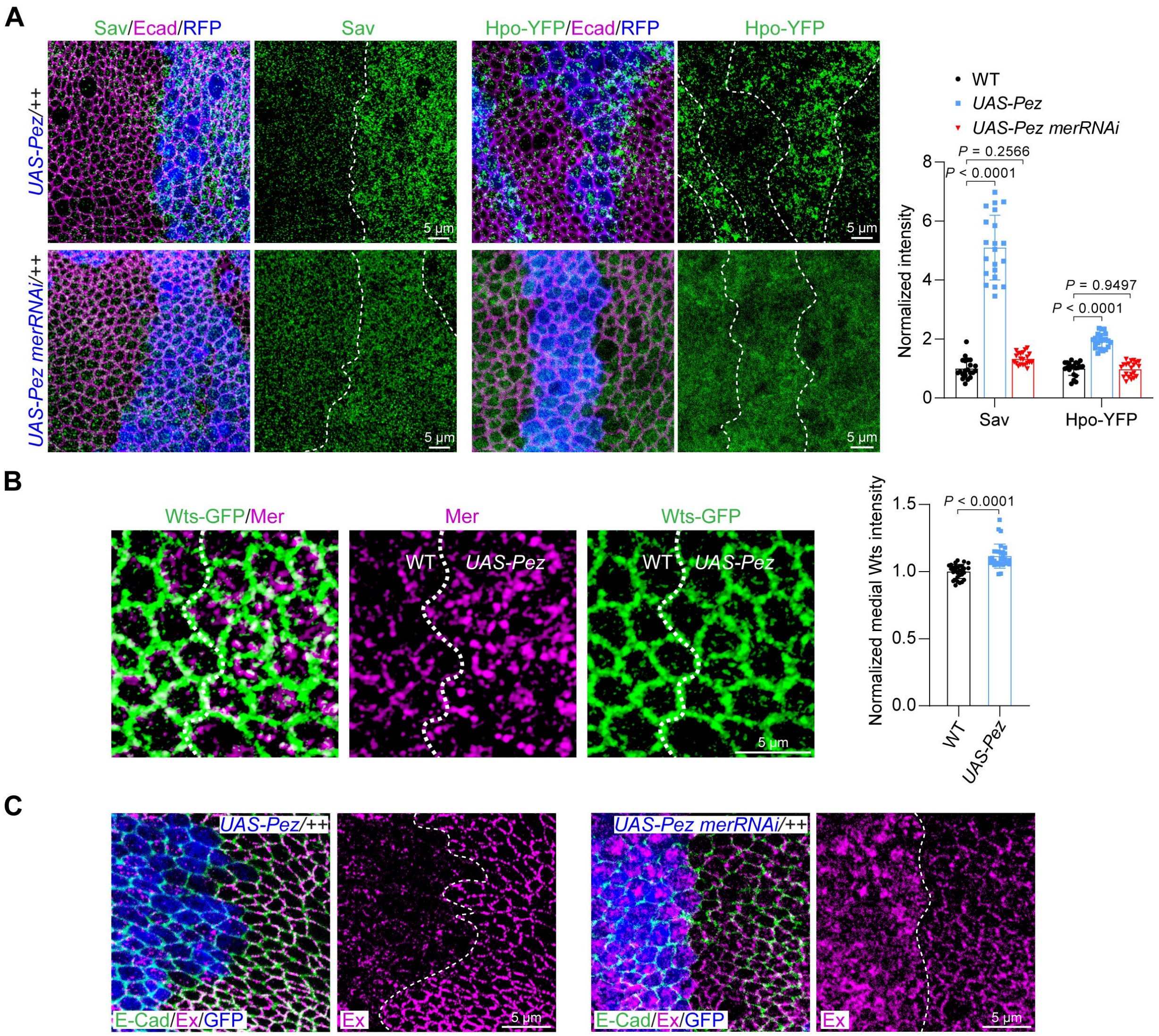

Fig. S9. Mer is required for Hippo pathway activation induced by Pez. (**A**) Wing discs containing RFP-positive Pez-overexpressing clones without (top) or with (bottom) *mer* RNAi were stained for Sav and Ecad (left) or Hpo-YFP and Ecad (right). Quantification of Sav and Hpo-YFP fluorescence intensity is shown to the right. Fluorescence intensity in cells in the indicated clones was normalized to the mean intensity in wild-type cells in the same disc. Note that *mer* knockdown diminished the apical accumulation of Sav and Hpo in Pez-overexpressing cells. Data are represented as mean ± s.d., n = 21, one-way ANOVA with Tukey's test. (**B**) A wing disc expressing Wts-GFP was stained for Mer. Pez was overexpressed in the posterior compartment using the *hh-Gal4* driver. Dashed lines mark the anterior-posterior compartment boundary. Quantification of medial apical Wts-GFP intensity was shown to the right. Data are represented as mean ± s.d., n = 30, unpaired two-tailed Student's t-test. (**C**) Wing discs

containing GFP-positive Pez-overexpressing clones without (left) or with (right) *mer* RNAi were stained for Expanded (Ex) and Ecad. Note that Pez overexpression decreased Ex expression in control but not *mer* knockdown cells.

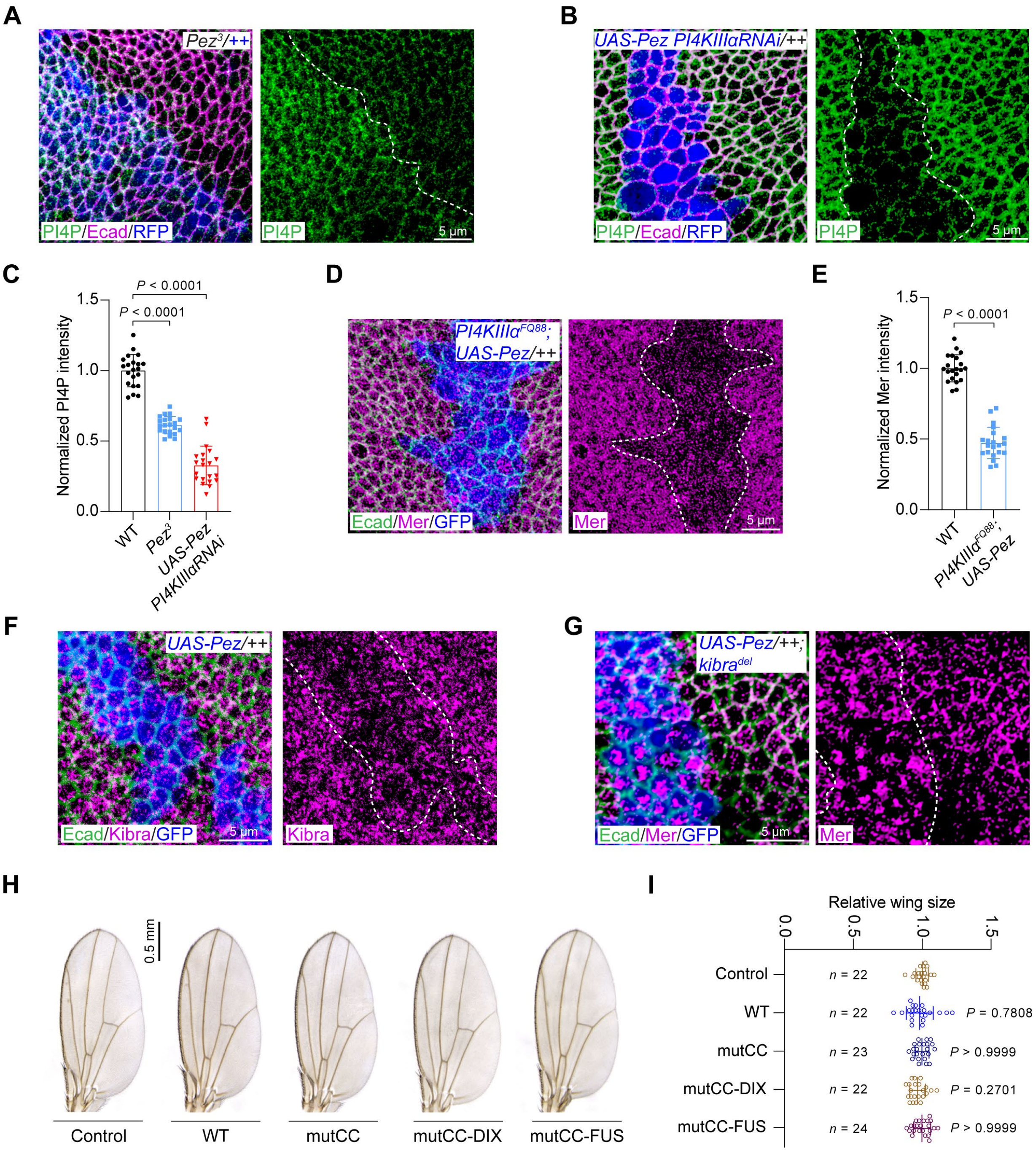

Fig. S10. Pez promotes the plasma membrane association of Mer by increasing abundance of PI4P. (**A**) A wing disc containing RFP-negative *Pez*^{3} clones and expressing the PI4P sensor GFP-2xOsh2-PH was stained for Ecad. (**B**) A wing disc expressing GFP-2xOsh2-PH and containing RFP-positive Pez-overexpressing clones with *PI4KIIIα* RNAi was stained for Ecad. (**C**) Quantification of GFP fluorescence intensity in (A) and (B) showing the reduction of PI4P in both clones. Data are represented as mean ± s.d., n = 21, one-way ANOVA with Dunnett's test.

(**D**) A wing disc containing GFP-positive *PI4KIIIα*^{FQ88} clones with Pez overexpression was stained for Ecad and Mer. (**E**) Quantification of Mer fluorescence intensity in (D) showing the reduction of Mer in *PI4KIIIα*^{FQ88} clones. Data are represented as mean ± s.d., $n = 21$, unpaired two-tailed Student's t-test. **(F)** Pez-overexpression does not induce Kibra accumulation. A wing disc containing GFP-positive Pez-overexpressing clones was stained for Kibra and Ecad. (**G**) Pez-induced medial apical Mer accumulation does not require Kibra. A wing disc containing GFP-positive *kibra*^{del} clones with Pez overexpression was stained for Ecad and Mer, showing the medial accumulation of Mer. (**H**) Adult wings carrying wild-type Mer or the indicated variants under the control of the tubulin promoter. (**I**) Quantitation of wing size in (H) showing that none of the tubulin-driven Mer transgenes caused visible wing phenotype by itself. Data are represented as mean ± s.d., one-way ANOVA with Dunnett's test.

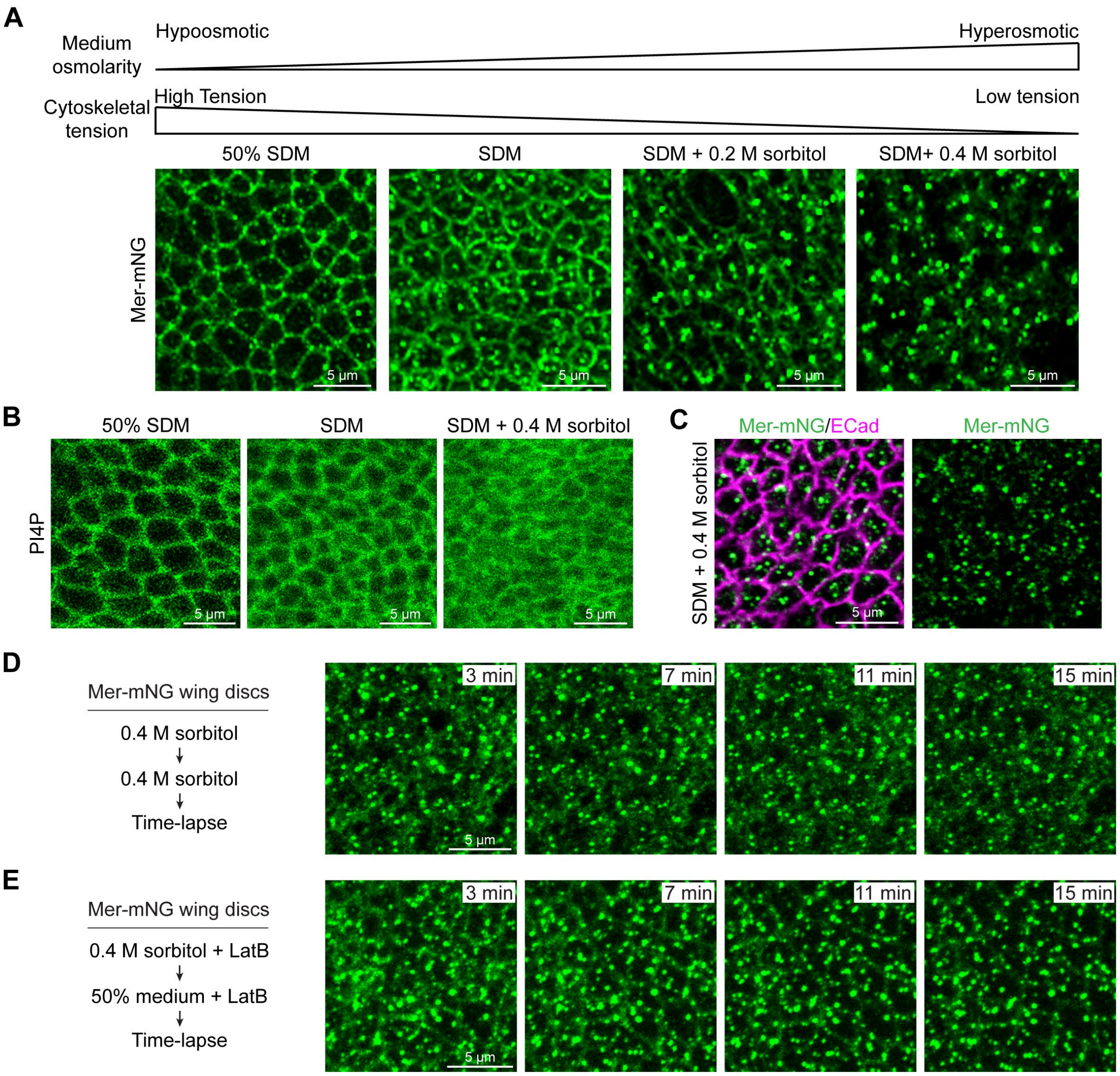

Fig. S11. Cytoskeletal tension promotes a physical state transition and disassembly of Mer condensates. (**A**) Wing discs expressing Mer-mNG were incubated in Schneider's *Drosophila* medium (SDM) of different osmolarity: 50% SDM (SDM diluted 1:1 with water), SDM, SDM with 0.2 M sorbitol, or SDM with 0.4 M sorbitol. Increasing osmolarity reduced cell cortical tension and promoted medial apical Mer condensation, while decreasing osmolarity increased cortical tension and promoted Mer junctional localization. (**B**) Wing discs expressing GFP-2xOsh2PH were incubated in hypoosmotic, normal or hyperosmotic medium for 30 minutes before imaging. (**C**) Wing discs expressing Mer-mNG were incubated in hyperosmotic medium for 30 minutes before Ecad staining, showing increased medial Mer condensates and the loss of junctional Mer (compare to the pattern of Mer-mNG in Fig. 2B). (**D**) Time-lapse images showing

the nearly identical punctate pattern of Mer during the 15-min time course. Wing discs expressing Mer-mNG were incubated in SDM containing 0.4 M sorbitol for 30 minutes before imaging in the same medium. (**E**) Time-lapse images showing that LatB treatment prevented the loss of medial Mer condensates induced by hypoosmotic stress. The experimental result was also presented in an abbreviated form in (Fig. 6E), but more time points are shown here.

**Figures**	**Panels**	***Genotypes***
1	A	*w^{1118}; tub-mer-mNG*
1	B	*w^{1118}; tub-mer-mNG*
2	A	*w^{1118}; tub-mer-mNG*
2	A	*w^{1118}; tub-mer^{mutCC}-mNG*
2	A	*w^{1118}; tub-mer^{mutCC}-DIX-mNG*
2	A	*w^{1118}; tub-mer^{mutCC}-FUS-mNG*
2	B	*w^{1118}; tub-mer-mNG*
2	B	*w^{1118}; tub-mer^{mutCC}-DIX-mNG*
2	B	*w^{1118}; tub-mer^{mutCC}-FUS-mNG*
2	E	*w^{1118}; tub-mer^{mutCC}-DIX-mNG*
2	E	*w^{1118}; tub-mer^{mutCC}-FUS-mNG*
3	D	*yw hsFlp Ubi-RFP FRT19A/mer^4 FRT19A*
3	D	*yw hsFlp Ubi-RFP FRT19A/mer^4 FRT19A; tub-mer-mNG/+*
3	D	*yw hsFlp Ubi-RFP FRT19A/mer^4 FRT19A; tub-mer^{mutCC}-mNG/+*
3	D	*yw hsFlp Ubi-RFP FRT19A/mer^4 FRT19A; tub-mer^{mutCC}-DIX-mNG/+*
3	D	*yw hsFlp Ubi-RFP FRT19A/mer^4 FRT19A; tub-mer^{mutCC}-FUS-mNG/+*
4	A	*yw hsFlp ubi-RFP FRT19A/PI4KIIIα^{FQ88} FRT19A; tub-GFP-2xOsh2-PH/+*
4	B	*yw hsFlp ubi-RFP FRT19A/PI4KIIIα^{FQ88} FRT19A*
4	D	*yw hsFlp UAS-GFP/+*; *Pez^3 FRT40A/tub-Gal80 FRT40A*; *tub-Gal4/+*
4	F	*yw hsFlp/+*; *Actin5C>CD2>Gal4/+*; *UAS-Pez/UAS-RFP*
4	G	*yw hsFlp/+; Actin5C>CD2>Gal4/+; UAS-Pez, tub-GFP-2xOsh2-PH/UAS-RFP*
4	I	*w^{1118}; tub-mer-mNG/nub-Gal4; UAS-Pez/+*
4	J	*w^{1118}; nub-Gal4/+; UAS-Pez/+*
4	J	*w^{1118}; tub-mer-mNG/nub-Gal4; UAS-Pez/+*
4	J	*w^{1118}; tub-mer^{mutCC}-mNG/nub-Gal4; UAS-Pez/+*
4	J	*w^{1118}; tub-mer^{mutCC}-DIX-mNG/nub-Gal4; UAS-Pez/+*
4	J	*w^{1118}; tub-mer^{mutCC}-FUS-mNG/nub-Gal4; UAS-Pez/+*
5	A-left	*w^{1118}; UAS-SqhEE/+; tub-GFP-2xOsh2-PH/hh-Gal4*

5	A-right	w^{1118}; *UAS-SqhEE/UAS-PI4KIIIα RNAi; tub-GFP-2xOsh2-PH/hh-Gal4*
5	C	*yw hsFlp/+; FRT42D* cpa^{69E}*/FRT42D ubi-GFP*
5	D-left	w^{1118}; *tub-mer-mNG nub-Gal4/+*
5	D-right	w^{1118}; *tub-mer-mNG nub-Gal4/UAS-*Sqh^{EE}
5	E	w^{1118}; *tub-mer-mNG nub-Gal4/UAS-*Sqh^{EE}
6	A, B, C, D, E	w^{1118}; *tub-mer-mNG*
6	F	w^{1118}; *tub-mer-mNG nub-Gal4/UAS-*Sqh^{EE}
7	A-left	w^{1118}; *tub-*mer^{mutCC}*-DIX-mNG nub-Gal4/+*
7	A-right	w^{1118}; *tub-*mer^{mutCC}*-DIX-mNG nub-Gal4/UAS-*Sqh^{EE}
7	B	w^{1118}; *tub-*mer^{mutCC}*-DIX-mNG*
7	C	w^{1118}; *tub-*mer^{mutCC}*-FUS-mNG*
7	E	w^{1118}; *tub-mer-mNG*
7	E	w^{1118}; *tub-*mer^{mutCC}*-DIX-mNG*
7	E	w^{1118}; *tub-*mer^{mutCC}*-FUS-mNG*
S1	A	w^{1118}; *tub-mer-mNG*
S1	C-left	w^{1118}; *tub-mer-mNG*
S1	C-middle	w^{1118}; *tub-mer-HA*
S1	C-right	w^{1118}
S1	D, G	w^{1118}; *tub-mer-mNG*
S1	I-left	w^{1118}; *tub-mer-mNG*
S1	I-right	w^{1118}
S2	A	w^{1118}; *nub-Gal4/+; UAS-kibra-GFP/+*
S6	A-left	w^{1118}; *tub-mer-HA*
S6	A-right	w^{1118}; *tub-*mer^{3A}*-HA*
S6	C	*yw hsFlp/+; FRT42D* $sktl^{\Delta 5}$*/FRT42D ubi-RFP; tub-GFP-PLCδ1-PH/+*
S6	D	*yw hsFlp/+; FRT42D* $sktl^{\Delta 5}$*/FRT42D ubi-RFP; tub-GFP-2xOsh2-PH/+*
S6	F, I	*yw hsFlp ubi-RFP FRT19A/*$PI4KIII\alpha^{FQ88}$ *FRT19A; tub-GFP-PLCδ1-PH/+*
S6	H	*yw hsFlp ubi-RFP FRT19A/*$PI4KIII\alpha^{FQ88}$ *FRT19A; tub-GFP-2xOsh2-PH/+*
S6	J	*yw hsFlp ubi-GFP FRT19A/*$PI4KIII\alpha^{FQ88}$ *FRT19A*

S6	K	*yw hsFlp ubi-GFP FRT19A/PI4KIIIα^{FQ88} FRT19A; tub-mer-HA/+*
S8	A	*yw hsFlp/+; FRT40A/l(2)cl-L3 ubi-GFP FRT40A*
S8	A	*yw hsFlp/+; ex^{e1} FRT40A/l(2)cl-L3 ubi-GFP FRT40A*
S8	A	*yw hsFlp/+; ex^{e1} Pez^{3} FRT40A/l(2)cl-L3 ubi-GFP FRT40A*
S8	A	*yw hsFlp/+; Pez^{3} FRT40A/l(2)cl-L3 ubi-GFP FRT40A*
S8	C	*w^{1118}; nub-Gal4/+*
S8	C	*w^{1118}; nub-Gal4/+; tub-mer-HA/+*
S8	C	*w^{1118}; nub-Gal4/+; tub-ex/+*
S8	C	*w^{1118}; nub-Gal4/+; UAS-mer RNAi/+*
S8	C	*w^{1118}; nub-Gal4/+; UAS-ex RNAi/+*
S8	C	*w^{1118}; nub-Gal4/+; UAS-Pez/+*
S8	C	*w^{1118}; nub-Gal4/+; UAS-Pez/tub-mer-HA*
S8	C	*w^{1118}; nub-Gal4/+; UAS-Pez/tub-ex*
S8	C	*w^{1118}; nub-Gal4/+; UAS-Pez/UAS-mer RNAi*
S8	C	*w^{1118}; nub-Gal4/+; UAS-Pez/UAS-ex RNAi*
S8	E	*yw hsFlp/+; Pez^{3} FRT40A/ubi-GFP FRT40A*
S8	E	*yw hsFlp/+; Pez^{3} FRT40A/ubi-GFP FRT40A; tub-mer-HA/+*
S9	A-upper left	*yw hsFlp/+; Actin5C>CD2>Gal4/+; UAS-Pez/UAS-RFP*
S9	A-bottom left	*yw hsFlp/+; Actin5C>CD2>Gal4/+; UAS-Pez UAS-mer RNAi/UAS-RFP*
S9	A-upper right	*yw hsFlp/+; Hpo-YFP/Actin5C>CD2>Gal4; UAS-Pez/UAS-RFP*
S9	A-bottom right	*yw hsFlp/+; Hpo-YFP/Actin5C>CD2>Gal4; UAS-Pez, UAS-mer RNAi/UAS-RFP*
S9	B	*w^{1118}; UAS-Pez, Wts-GFP/hh-Gal4*
S9	A-left	*yw hsFlp/+; Actin5C>CD2>Gal4/+; UAS-Pez/UAS-GFP*
S9	A-right	*yw hsFlp/+; Actin5C>CD2>Gal4/+; UAS-Pez UAS-mer RNAi/UAS-GFP*
S10	A	*yw hsFlp/+; Pez^{3} FRT40A/ubi-RFP FRT40A; tub-GFP-2xOsh2-PH/+*
S10	B	*yw hsFlp/+; UAS-PI4KIIIα RNAi/Actin5C>CD2>Gal4; UAS-Pez, tub-GFP-2xOsh2-PH/UAS-RFP*
S10	D	*w* hsFlp tub-Gal80 FRT19A/PI4KIIIα^{FQ88} FRT19A; UAS-GFP/+; UAS-Pez/tub-Gal4*
S10	F	*yw hsFlp/+; Actin5C>CD2>Gal4/+; UAS-Pez/UAS-GFP*
S10	G	*w* hsFlp; UAS-GFP/tub-Gal4; UAS-Pez, kibra^{delta} FRT82B/tub-Gal80 FRT82B*

S10	H	*w^{1118}; nub-Gal4/+*
S10	H	*w^{1118}; nub-Gal4/tub-mer-mNG*
S10	H	*w^{1118}; nub-Gal4/tub-mer^{mutCC}-mNG*
S10	H	*w^{1118}; nub-Gal4/tub-mer^{mutCC}-DIX-mNG*
S10	H	*w^{1118}; nub-Gal4/tub-mer^{mutCC}-FUS-mNG*
S11	A, B, C, D, E	*w^{1118}; tub-mer-mNG*

Table S1. A list of genotypes for all experimental crosses.
